# Phytochemistry reflects different evolutionary history in traditional classes versus specialized structural motifs

**DOI:** 10.1038/s41598-021-96431-3

**Published:** 2021-08-26

**Authors:** Kathryn A. Uckele, Joshua P. Jahner, Eric J. Tepe, Lora A. Richards, Lee A. Dyer, Kaitlin M. Ochsenrider, Casey S. Philbin, Massuo J. Kato, Lydia F. Yamaguchi, Matthew L. Forister, Angela M. Smilanich, Craig D. Dodson, Christopher S. Jeffrey, Thomas L. Parchman

**Affiliations:** 1 Program in Ecology, Evolution, and Conservation Biology, University of Nevada, Reno, NV 89557 USA; 2 Department of Biology, University of Nevada, Reno, NV 89557 USA; 3 Hitchcock Center for Chemical Ecology, University of Nevada, Reno, NV 89557 USA; 4 Department of Biological Sciences, University of Cincinnati, Cincinnati, OH 45221 USA; 5 Sección Invertebrados, Museo Ecuatoriano de Ciencias Naturales, Quito, Ecuador; 6 Department of Chemistry, University of Nevada, Reno, NV 89557 USA; 7 Department of Fundamental Chemistry, Institute of Chemistry, University of São Paulo, São Paulo, Brazil

**Keywords:** Phylogenetics, Chemical ecology

## Abstract

Foundational hypotheses addressing plant–insect codiversification and plant defense theory typically assume a macroevolutionary pattern whereby closely related plants have similar chemical profiles. However, numerous studies have documented variation in the degree of phytochemical trait lability, raising the possibility that phytochemical evolution is more nuanced than initially assumed. We utilize proton nuclear magnetic resonance (^1^H NMR) data, chemical classification, and double digest restriction-site associated DNA sequencing (ddRADseq) to resolve evolutionary relationships and characterize the evolution of secondary chemistry in the Neotropical plant clade Radula (*Piper*; Piperaceae). Sequencing data substantially improved phylogenetic resolution relative to past studies, and spectroscopic characterization revealed the presence of 35 metabolite classes. Metabolite classes displayed phylogenetic signal, whereas the crude ^1^H NMR spectra featured little evidence of phylogenetic signal in multivariate tests of chemical resonances. Evolutionary correlations were detected in two pairs of compound classes (flavonoids with chalcones; *p*-alkenyl phenols with kavalactones), where the gain or loss of a class was dependent on the other’s state. Overall, the evolution of secondary chemistry in Radula is characterized by strong phylogenetic signal of traditional compound classes and weak phylogenetic signal of specialized chemical motifs, consistent with both classic evolutionary hypotheses and recent examinations of phytochemical evolution in young lineages.

## Introduction

Plant secondary chemistry affects plant–herbivore interactions at various stages throughout an insect’s lifespan: mixtures of compounds can shape adult oviposition preferences^1^, specific chemical compounds can stimulate larval feeding^2^, specific chemotypes can deter insect herbivores via toxicity or physiological disruptions^3^, and sequestered metabolites can alter immune function against natural enemies^4^. Plants capable of developing novel chemical defenses are hypothesized to accrue higher fitness due to enemy release^5^, potentially resulting in the diversification of plant lineages with conserved chemical phenotypes (the escape and radiate hypothesis^6^). Coevolutionary hypotheses and plant defense theory have yielded clear predictions that herbivory, additional trophic interactions, and resource availability shape the evolution of plant defenses, including secondary metabolites^7,8^. However, an evolutionary response to these biotic and abiotic pressures could be complex and highly context-dependent.

Due in part to the enzymatic complexity of metabolic biosynthesis, phylogenetic conservatism is the null hypothesis for the evolution of plant secondary chemistry^9,10^. Indeed, expectations of phylogenetic conservatism appear to hold at deep evolutionary scales; for example, the family Solanaceae is characterized by the presence of tropane alkaloids^11^, though they are consistently present in only 3 of 19 tribes (Datureae, Hyoscyameae, Mandragoreae) and sporadically found elsewhere^12^. Further, recent work suggests that classes of secondary metabolites are more likely to be phylogenetically conserved in large seed plant clades (e.g., eudicots and superasterids) than at lower taxonomic scales (e.g., orders and families)^13^. At shallower scales, numerous studies provide evidence for evolutionary lability in chemical traits within genera^7,14–16^, suggesting that surveys of phytochemical variation within young plant lineages might yield variable perspectives on the evolution of secondary chemistry. Adding further complexity, many studies have found evidence for strong evolutionary associations among chemical classes^16,17^. For example, Johnson et al.^18^ found a strong positive correlation between flavonoids and phenolic diversity and a strong negative correlation between ellagitannins and flavonoids across a phylogeny of 26 evening primroses (*Oenethera*: Onagraceae). Such associations are relevant because they may reflect evolutionary constraints, and their causes may be varied. For example, positive associations may be associated with chemical defense syndromes^9,19^ or synergistic effects of multiple classes on herbivore deterrence^20^. Alternatively, negative associations might be consistent with evolutionary tradeoffs or at least different optima in defense space^18,19^. By leveraging advances in organic chemistry and genomics, we stand to increase metabolomic and phylogenetic resolution to provide novel insight into the evolution of phytochemistry.

Recent advances in chemical ecology have improved perspectives on phytochemical diversity across a broad range of taxonomic groups and metabolite classes^21,22^. High throughput processing of plant tissue, rapid advances in spectroscopy, and improved ordination and network analyses have enabled characterization of metabolomic variation across plant communities^10,15,22–24^ and stand to enhance our understanding of phytochemical evolution across taxonomic scales^21^. Additionally, structural spectroscopic approaches like ^1^H NMR can provide improved resolution of structural variation across a wide range of metabolite classes. Selection on the plant metabolome is inherently multivariate, arising from diverse herbivore communities and environmental conditions^10,25^, and even relatively small structural changes can impart disproportionate shifts in bioactivity. Thus, approaches that capture a larger proportion of the structural variation underlying phytochemical phenotypes could be well suited to addressing hypotheses concerning evolutionary patterns.

Next-generation sequencing data has reinvigorated phylogenetic analyses of traditionally challenging groups characterized by recent or rapid diversification^26^. Reduced representation DNA sequencing approaches [e.g., ddRADseq; genotyping-by-sequencing (GBS)] have been increasingly utilized in phylogenetic studies due to their ability to effectively sample large numbers of orthologous loci throughout the genomes of non-model organisms without the need for prior genomic resources^27^. Nearly all such studies have reported increased topological accuracy and support compared with past phylogenetic inference based on smaller numbers of Sanger-sequenced loci^28,29^, especially when applied to diverse radiations^30,31^. While reduced representation approaches have clear phylogenetic utility at relatively shallow time scales, they have also performed well for moderately deep divergence^29,32^.

*Piper* (Piperaceae) is a highly diverse, pantropical genus of nearly 2,600 accepted species^33^, with the highest diversity occurring in the Neotropics^34^. Chemically, *Piper* is impressively diverse^35–37^: chemical profiling in a modest number of taxa has yielded 667 different compounds from 11 distinct structural classes thus far^35,36,38,39^. This phytochemical diversity has likely contributed to the diversification of several herbivorous insect lineages that specialize on *Piper*, including the geometrid moth genus *Eois*^40^ (Larentiinae). Furthermore, phytochemical diversity in *Piper* communities has been shown to shape tri-trophic interactions and the structure of tropical communities^36,39,41^. As a species-rich genus with abundant and ecologically consequential phytochemical diversity, *Piper* represents a valuable system for understanding how complex diversification histories underlie the evolution of phytochemical diversity.

*Piper* is an old lineage (~ 72 Ma), yet most of its diversification occurred in the Neotropics during the last 30–40 My following Andean uplift and the emergence of Central America^34,42^. The largest clade of *Piper*, Radula, exemplifies this pattern, as much of its extant diversity (~ 450 species) arose relatively recently during the Miocene^34^. Such bouts of rapid and recent diversification have limited the efficacy of traditional Sanger sequencing methods to resolve the timing and tempo of diversification in *Piper*^42,43^. Past phylogenetic analyses utilizing Sanger-sequenced nuclear and chloroplast regions have consistently inferred eleven major clades within *Piper*; however, phylogenetic resolution within these clades has been elusive^42–45^. Phylogenetic inference based on genome-wide data spanning a range of genealogical histories should facilitate an understanding of evolutionary patterns of phytochemical diversity in *Piper* and their consequences for plant–insect codiversification.

We leveraged complementary phylogenomic, metabolite classification, and ^1^H NMR data sets to generate a *Piper* phylogeny and explore the evolution of secondary chemistry within the largest *Piper* clade (Radula). We used reduced representation sequencing (ddRADseq) to generate genome-wide data for 71 individuals, spanning eight *Piper* clades but focusing on Radula, for phylogenetic analyses. Due to its ability to characterize subtle structural variation across a wide range of compound classes, we used nuclear magnetic resonance (^1^H NMR) spectroscopy to quantify phytochemical diversity in the same individuals. Our goals were to: 1) resolve the evolutionary relationships within the Radula clade of *Piper* included in this study; 2) characterize metabolomic variation across the genus and within Radula in particular; and 3) quantify the strength of phylogenetic signal and test for evolutionary associations in Radula secondary chemistry. Because secondary chemistry is an emergent composite phenotype of many traits that can evolve semi-independently, we expected to detect mixed strengths of phylogenetic signal and strong associations among a subset of traits over evolutionary time.

## Results

### Phylogenetic analyses

After contaminant filtering and demultiplexing, we retained ~ 313 million Illumina reads for phylogenetic analyses. Initial clustering, variant calling, and filtering assembled reads into 362,169 ddRADseq loci. There was a high proportion of missing data, presumably due to allelic dropout increasing with high levels of divergence among *Piper* clades. For Bayesian phylogenetic inference, we mitigated the influence of missing data by removing loci absent in > 30% of samples. The final dataset for phylogenetic analysis consisted of 641 ddRADseq loci (~ 86 bp in length each) that housed 9113 genetic variants (51% parsimony informative). Aligned loci were concatenated into a nexus alignment with missing data at 18.9% of sites.

**Figure 1:**
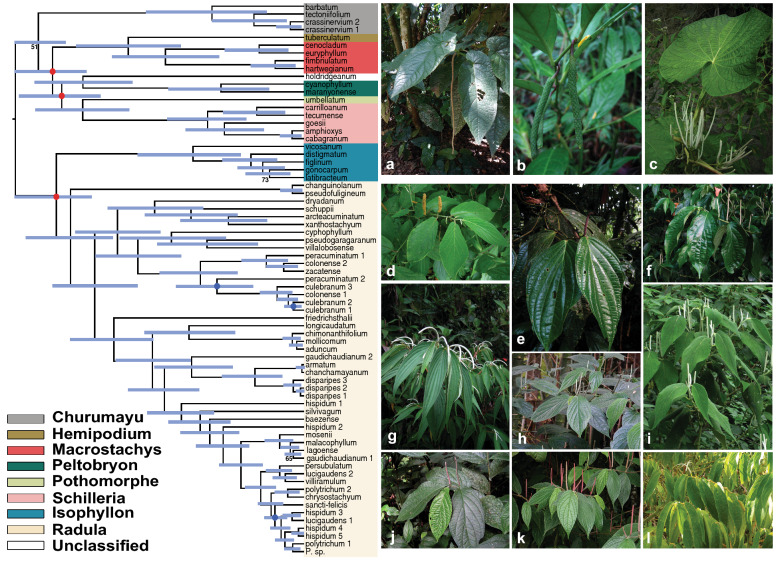
Maximum clade credibility tree of 48 samples from the Radula clade of *Piper* and 23 outgroup species inferred with a Bayesian analysis of 641 concatenated ddRADseq loci (55,298 base pairs) comprising 9113 genetic variants (of which 4674 are parsimony informative). The outgroup taxa were sampled across multiple *Piper* clades: Churumayu, Isophyllon, Hemipodium, Macrostachys, Peltobryon, Pothomorphe, and Schilleria. All nodes are supported by at least 95% posterior support except where noted with circles or labels. Blue circles indicate support values between 85 and 95%. Red circles indicate support values between 75 and 85%. Three nodes with less than 75% posterior support were given numerical support values. Blue bars at each node denote the 95% highest posterior density interval on relative node ages. The photos to the right of the tree showcase a sample of *Piper* diversity, including a few of the species which were included in this study: (**a**) *Piper hillianum* (Macrostachys), (**b**) *P. acutifolium* (Peltobryon), (**c**) *P. umbellatum* (Pothomorphe), (**d**) *P. pseudofuligineum* (Radula), (**e**) *P. concepcionis* (Radula), (**f**) *P. disparipes* (Radula), (**g**) *P. friedrichsthalii* (Radula), (**h**) *P. dilatatum* (Radula), (**i**) *P. bredemeyeri* (Radula), (**j**) *P. immutatum* (Radula), (**k**) *P. erubescentispicum* (Radula), and (**l**) the widespread and often weedy *P. aduncum* (Radula). (Photo credits: E. J. Tepe).

Bayesian phylogenetic analysis of ddRADseq data resolved eight major Neotropical *Piper* clades with high posterior support (Fig. 1). While past phylogenetic studies supported the monophyly of seven of these eight clades (Macrostachys, Radula, Peltobryon, Pothomorphe, Hemipodium, Isophyllon, and Schilleria)^34,43^, our analysis resolved an additional clade, Churumayu. Notably, Isophyllon and Churumayu were highly supported, monophyletic clades and not nested within Radula, as was inferred in previous analyses^43^. Contrary to previous phylogenetic hypotheses of *Piper*^34,43^, our analysis might suggest Churumayu is the most basal clade, but we caution that this node had very low posterior support (51%). Intrageneric relationships below the clade level were highly resolved, with nearly all nodes exhibiting greater than 95% posterior support, including within the diverse Radula clade (Fig. 1). Our phylogenetic hypothesis for Radula indicates three species (*P. hispidum*, *P. colonense*, *P. lucigaudens*) may be paraphyletic.

### Phytochemical diversity in *Piper*

**Figure 2:**
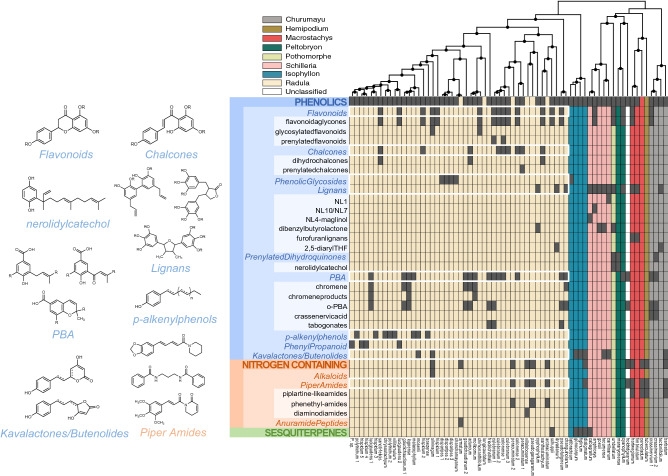
Patterns of chemical variation are displayed for individuals in this study. Taxa comprise the columns of the matrix and are ordered according to their inferred phylogenetic relationships. Groups of columns are colored according to their designated *Piper* clade. Black circles within the phylogenetic tree designate nodes with posterior support values greater than 85%. Each row of the matrix represents a metabolite class that was detected from ^1^H NMR and MS-based methods, and dark grey cells indicate the presence of that class in that taxa. Classes are hierarchically nested; capitalized font signifies the three classes at the highest level (and coarsest resolution), italicized font signifies the intermediate level, and black font signifies the lowest level (and highest resolution). Rows outlined in white indicate traits that were analyzed for phylogenetic signal in Radula. To the left of the matrix are representative compounds for a subset of metabolite classes that were detected in our samples.

All but four individuals included in the inferred *Piper* tree were successfully chemically extracted and profiled. Nearly all common compound classes that have been previously reported in *Piper*^46^ were observed from our compound characterization analysis (see Table S2). This analysis revealed the presence of broad metabolite classes that are ubiquitous across plant families (e.g., lignans, flavonoids/chalcones, etc.) as well as classes that are specifically common in *Piper* (e.g., amides) (Fig. 2, Table S2). Specific compound characterization revealed genus specific compounds and compound classes (piplartine, cenocladamide, crassinervic acid, kava lactones), as well as metabolites that are more rarely reported in plants (putrescine diamides, nerolidyl catechol, alkenyl phenols, anuramide peptides) (Fig. 2, Table S2). Alternative methods, such as sampling across a species’ ontogeny, sampling reproductive parts or roots, and storing freshly collected tissue in methanol rather than air drying would add to a more comprehensive picture of variation in phytochemical diversity across and within species, but our sampling was standardized to allow for initial comparisons across species, some of which were collected in remote regions.

### Metabolite phylogenetic signal and evolutionary associations

We recovered 35 metabolite classes, of which only eight were sufficiently present across our taxa to afford tests of phylogenetic signal and correlated evolution. For all eight metabolite classes, estimates of D did not deviate from a null distribution generated under a scenario of Brownian motion (Table 1), consistent with phylogenetic signal. Two of the eight traits, phenolic glycosides and lignans, exhibited strong phylogenetic signal (D < 0), while the remaining six traits exhibited weak phylogenetic signal (0 < D < 1). Further, all but one of the metabolite classes had observed values of D that differed from a null distribution generated under a phylogenetic randomness scenario (Table 1). The mean of the observed D estimates for the metabolite classes was 0.06, with the largest D statistic observed for the chalcone class (D = 0.62) and the smallest observed for the phenolic glycosides (D = − 1.18) (Table 1).

**Table 1:** Estimates of phylogenetic signal (D)^57^ for a subset of metabolite classes (see “Methods” for explanation of subset).

Metabolite class	Observed D	$\Sigma {d}_{obs}$	Randomness (H_0_: D = 1)		Brownian (H_0_: D = 0)	
			$Mean(\Sigma {d}_{r})$	*P*	$Mean(\Sigma {d}_{b})$	*P*
Flavonoids	0.49	14.18	17.56	0.012	11.01	0.093
Chalcones	0.62	11.59	13.33	0.095	8.79	0.088
Phenolic glycosides	−1.18	3.11	7.01	0.000	5.19	0.950
Lignans	−0.02	4.16	5.47	0.036	4.19	0.564
PBA	0.22	12.40	17.51	0.001	10.96	0.293
*p*-alkenyl phenols	0.33	9.47	12.30	0.010	8.19	0.265
Kavalactones/butenolides	0.02	5.17	6.99	0.027	5.18	0.504
Piper amides	0.1	5.37	7.00	0.033	5.18	0.482

To ask whether traits evolved under scenarios of Brownian motion (D = 0) or phylogenetic randomness (D = 1), observed values of D were compared to null distributions of D modeled under each scenario.

**Figure 3:**
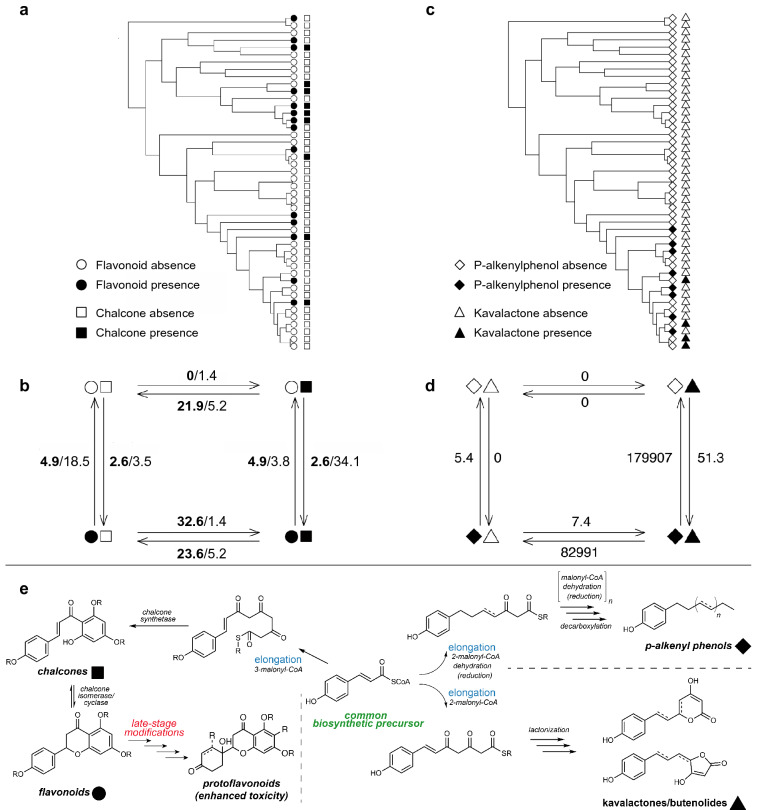
Evolutionary associations were detected in two pairs of traits according to Pagel’s test^76^ of correlated evolution: (1) flavonoids and chalcones and (2) *p*-alkenyl phenols and kavalactones/butenolides. Filled shapes indicate presences and unfilled shapes indicate absences of flavonoids (circles), chalcones (squares), *p*-alkenyl phenols (diamonds), and kavalactones/butenolides (triangles), respectively. The shapes used in the phylogenetic plots (**a,c**) are repeated below (**b,d**) to depict four states comprising all combinations of presences and absences in the pair of traits. Arrows represent transition rates between states. (**b**) As both models of contingent change provided good fits to the flavonoid and chalcone data, both sets of transition rates are displayed, with the first set of values (bolded) corresponding to the best supported model (chalcone evolution contingent on flavonoid state) and the second set of values corresponding to the alternative contingency model (flavonoid evolution contingent on chalcone state). (**d**) The best fit model to the *p*-alkenyl phenol and kavalactone/butenolide data was one of interdependent evolution, where *p*-alkenyl phenol evolution is dependent on the state at the kavalactone/butenolide trait, and vice versa. Panel (**e**) illustrates the enzymatic processes and branch points along biosynthetic pathways that give rise to the four classes of metabolites. Chalcones are immediate biosynthetic precursors of flavonoids, where the inherent reactivity of the chalcone moiety permits cyclization to the flavonoid scaffold. Subtle structural changes to the flavonoid scaffold caused by late-stage oxidation can produce protoflavonoids, a rare class of metabolite with potent cytotoxic activity. In contrast, the pathways of *p-*alkenyl phenols and kavalactones diverge much earlier and embark on distinct chain elongation pathways that lead to long-chain lipophilic substituent characteristic of the *p*-alkenyl phenols in one case, and lactones (kavalactones and butenolides) in the other case.

Of the 28 pairwise tests of correlated evolution, only two were significant based on a significance level of 0.05. Evidence for correlated evolution was detected in two pairs of metabolite classes: (1) flavonoids and chalcones; and (2) *p*-alkenyl phenols and kavalactones/butenolides. For the first pair of traits, a model of contingency in which changes in chalcones depend on the state of flavonoids provided the best fit to the data (Table 2). In this model, when flavonoids are present, chalcone gains are 1.4 times more probable than chalcone losses; however, when flavonoids are absent, chalcone losses are much more probable than chalcone gains (Fig. 3). The alternative contingency model for this pair of traits (i.e., changes in flavonoids depend on the state of chalcones) was also a good fit to the data (Table 2). According to this model, when chalcones are present, flavonoid gains are approximately nine times more probable than flavonoid losses. Alternatively, when chalcones are absent, flavonoid losses are approximately five times more probable than flavonoid gains (Fig. 3). For the second pair of traits, *p*-alkenyl phenols and kavalactones/butenolides, the best fit model was one of interdependent evolution in which changes in *p*-alkenyl phenol depend on the state of kavalactones/butenolides, and vice versa (Table 2). When kavalactones/butenolides are present, *p*-alkenyl phenol transitions are more probable than when they are absent, with the loss of *p*-alkenyl phenols being much more probable than the gain of *p*-alkenyl phenols under both scenarios. Alternatively, when *p*-alkenyl phenols are present, the loss of kavalactones/butenolides is extremely probable relative to the gain of kavalactones/butenolides, which is rarely observed. When *p*-alkenyl phenols are absent, kavalactones/butenolides are rarely gained or lost (Fig. 3).

**Table 2:** Correlated evolution was detected in two pairs of metabolite classes with Pagel’s method^76^: (1) chalcones and flavonoids; and (2) kavalactones/butenolides and *p*-alkenyl phenols.

Comparison	Model	AIC	Δ AIC	AIC weight
Chalcones, flavonoids	Chalcones contingent on flavonoids	95.58	0	0.51
	Flavonoids contingent on chalcones	96.02	0.44	0.41
	Interdependent evolution	99.84	4.26	0.06
	Independent evolution	102.46	6.88	0.02
Kavalactones/butenolides, *p*-alkenyl phenols	Interdependent evolution	62.35	0	0.95
	Kavalactones/butenolides contingent on *p*-alkenyl phenols	69.65	7.29	0.03
	*p*-alkenyl phenols contingent on kavalactones/butenolides	70.61	8.26	0.02
	Independent evolution	71.57	9.22	0.01

A model comparison framework was employed to evaluate four potential models of trait evolution using AIC: interdependent evolution (transition rate in one trait depends on state at another, and vice versa); contingent change (transition rate in one trait depends on state at another, but not the converse); and independent evolution.

### Phylogenetic signal in high-dimensional metabolomic data

**Figure 4:**
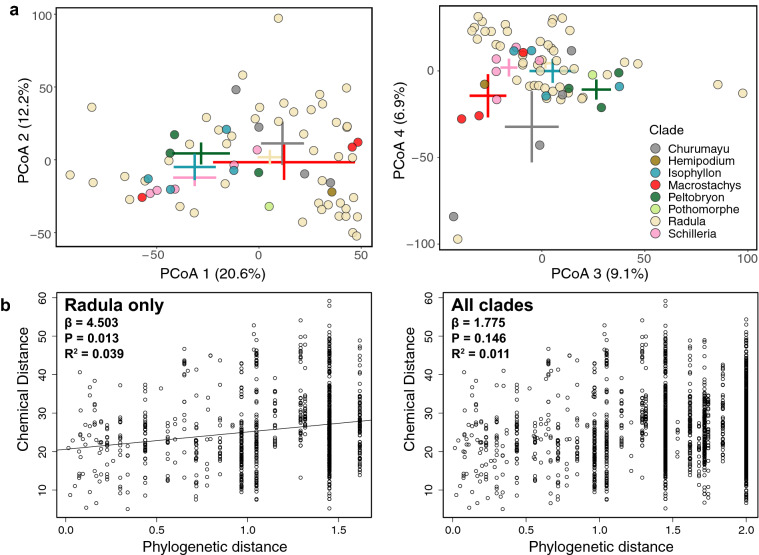
(**a**) Chemospace of all 67 *Piper* samples constructed with the crude ^1^H NMR data across 263 peaks. Point colors were chosen according to clade designation as portrayed in the phylogenetic tree in Fig. 1. A legend is provided in the rightmost PCoA plot. Error bars depict estimates of ordination mean + /− 3 standard errors for each clade. (**b**) MRM analyses recovered a significant positive relationship between phylogenetic and chemical distances calculated among samples from the Radula clade (left), but did not recover a significant relationship when calculated among all samples from all clades (right).

While the eight metabolite classes uniformly exhibited at least moderate levels of phylogenetic signal, evidence for phylogenetic signal in multivariate analyses of the crude ^1^H NMR data was largely absent. PCo axes 1 & 2 and 3 & 4 explained 32.8% and 16.0% of variance in the ^1^H NMR data, respectively, but showed little clustering by clade (Fig. 4a). Permutational multivariate analyses of variance were not significant for combinations of either PCo 1 & 2 (*P* = 0.407) nor 3 & 4 (*P* = 0.142), suggesting that different clades do not form distinct clusters in chemospace based on their ^1^H NMR spectra.

According to the MRM models, phylogenetic distance significantly predicts phytochemical distance within Radula (*β* = 4.503, *P* = 0.013) but not across all clades (*β* = 1.775, *P* = 0.146) (Fig. 4b). It is important to note that the proportion of variance explained by the significant MRM model is low (*R*^2^ = 0.039), suggesting that the majority of variation in NMR data cannot be explained by phylogenetic distance.

Analyses with the generalized *K* statistic (*K*_mult_) indicated lower levels of phylogenetic signal in the metabolomic data than expected under a Brownian motion model of evolution for *Piper* generally (*K*_mult_ = 0.1606, *P* = 0.001) and for Radula specifically (*K*_mult_ = 0.1803, *P* = 0.001). Still, the observed *K*_mult_ was higher than all *K*_mult_ values obtained with permutations of the ^1^H NMR dataset (Fig. S1). Additionally, few *K*_mult_ tests of the permuted data yielded significant *P*-values (4.4% of permutations), indicating that the estimate we observed, though subtle and lower than Brownian motion expectations, was real and not a statistical artifact of zero-inflation in the data.

## Discussion

*Piper* is a hyper-diverse lineage in which phytochemical diversity has influenced evolutionary and ecological processes and shaped complex tropical communities^15,39^. However, limitations in both the degree of phylogenetic resolution and the understanding of phytochemical diversity in this group have precluded analyses of phylogenetic signal and correlated evolution of phytochemistry. Phylogenies inferred here with ddRADseq data substantially improved resolution and support compared to past studies of *Piper*, which were limited by interspecific variation in small numbers of Sanger-sequenced loci^34,42,43^. Although the data set did not include members from all previously recognized groups, analyses resolved eight monophyletic Neotropical *Piper* clades, seven of which have been inferred in previous analyses of the genus based on chloroplast psbJ-petA and nrITS^34,43^. Two of the eight clades, Churumayu and Isophyllon, had been previously nested within Radula^43^; however, our results suggest that they are independent monophyletic lineages (Fig. 1). Despite low support for several deep divergences, the phylogeny inferred here had strong resolution and support for recent relationships, including within Radula (Fig. 1), consistent with other recent reduced representation sequencing studies that have generated high quality phylogenies at shallow time scales^28,31,32^. However, a potential limitation of such sequencing designs may include the recovery of fewer loci shared by more distantly related samples due to allelic dropout^47^. It is possible that allelic dropout, potentially exacerbated by strict filtering based on missing data, led to weak support values for deep splits in the phylogeny, many of which occurred early in the history of the Neotropical *Piper* lineage^34^. Nonetheless, the resulting subset of data (641 loci; 9113 SNPs) was sufficient for inferring a largely resolved phylogeny, highlighting the potential promise of reduced representation sequencing for resolving evolutionary histories even in groups spanning moderately deep divergence. Although our sampling was limited to 44 of 450 estimated species within Radula, the extent of sampling is a substantial improvement over past phylogenetic analyses for the group^42,43^.

Comparative studies have taken diverse approaches to analyzing metabolomic data, each providing a unique perspective on the evolution of specialized metabolites^10,24^. Here, we first characterized the presence/absence of 35 metabolite classes commonly used to categorize plant secondary compounds that are hierarchically nested into three levels of structural resolution. Specific categories at the lowest level of the hierarchy, representing specialized structural motifs or specific molecules, were rare across species and precluded tests of phylogenetic signal and correlated evolution at our level of taxonomic sampling (Fig. 2). Despite not being able to test for phylogenetic signal, clustering is evident for more specific categories, such as crassinervic acid and prenylated flavonoids, which are only present in small subclades but include particularly effective defenses^36,46^. Alternatively, broader metabolite classes at intermediate and high positions in the hierarchy that are directly tied to fundamental secondary metabolite biosynthetic pathways were more abundant across species and exhibited moderately high levels of phylogenetic signal across Radula (Table 1, Fig. 2). This pattern may be expected if initial biosynthetic steps are conserved over longer evolutionary scales, permitting the abundance of broad chemical classes, yet later stage modifications of these core structures are more evolutionarily labile, causing structural similarity to be low even among related species. Flavonoids are a good example of this pattern, with pathways that form the flavonoid scaffold being very conserved, as they are catalyzed by modified enzymes from ubiquitous metabolic pathways, but then subsequent biosynthetic steps (e.g., those catalyzed by p450 enzymes) modify these scaffolds^48^, yielding unique molecules towards the tips of evolutionary trees (Fig. 3E). For example, late-stage modification of common flavonoid scaffolds can result in the production of non-aromatic protoflavonoids. These compounds rarely occur across the plant kingdom and have only recently been found in one species of *Piper*^49^, but this type of subtle structural modification that leaves most of the flavonoid scaffold intact dramatically enhances the cytotoxic properties compared to that of the parent flavonoid^50,51^.

One key prediction from the escape and radiate hypothesis is that adaptive defensive traits should be phylogenetically conserved within the lineage they evolved, but this prediction has mostly been evaluated with broad classes of secondary metabolites at high taxonomic scales^6,13,48^ rather than specific compounds in recent diversifications^7,10,16^. A growing number of studies conducted at shallow evolutionary scales suggests low phylogenetic signal in many chemical traits^14,15,18^. While evidence for low phylogenetic signal is often attributed to high evolutionary rates (i.e., evolutionary lability), simulations under various evolutionary processes and conditions indicate that the relationship between phylogenetic signal and rate of trait evolution is not necessarily straightforward, and evidence for low phylogenetic signal is not an indication of any single evolutionary process^52^. Nonetheless, understanding how phylogenetic signal responds to variation in phylogenetic scale is informative in a comparative sense, especially among different traits or classes of traits generated with different levels of analytical resolution. Phylogenetic signal is also a useful starting point for developing insights into the drivers of herbivorous insect radiations, as codiversification in many of these lineages is structured in part by chemical defense and biotic interactions^40,53^. Our results are generally consistent with the predictions of moderately strong signal for broad classes of compounds, as well as the lack of signal for specific structures captured by ^1^H NMR data.

The ^1^H NMR data address a different set of hypotheses than data from categorization of individual molecules—peaks represent resonances associated with particular molecular structures rather than individual compounds, and the chemical shift (frequency), shape, and abundance of these resonances are extremely sensitive to subtle structural changes. ^1^H NMR spectroscopy easily detects a great range and subtle differences in compositional and structural complexity, including increasing size, asymmetry and oxidation states, that might be predicted to evolve in response to divergent selection across plant populations responding to different suites of enemies^22^. Low levels of phylogenetic signal in the ^1^H NMR data is also likely due to the fact that many molecular features of small defensive molecules have potentially evolved in a convergent manner across *Piper*, such as the kavalactones, *p*-alkenyl phenols, piplartine, oxidized prenylated benzoic acids, chromenes, anuramide peptides, and phenethyl amides.

There are numerous limitations that could affect estimates of phylogenetic signal in comparative studies^54^ that are relevant to the analyses presented here. First, incomplete taxon sampling likely influenced our results to some degree, but sampling was conducted randomly, and the probability that a particular species was sampled was unlikely related to any aspect of its chemical phenotype^55^. Low sampling proportion in clades other than Radula may have reduced our power to detect phylogenetic signal across all our sampled clades^55^ (Fig. 4b). However, despite only sampling approximately 10% of the Radula clade of *Piper*, our sample size should provide sufficient power to infer phylogenetic signal in this clade if present^56,57^ (Fig. 4b). Second, while topological errors and small sample size may have reduced our power to detect phylogenetic signal at deeper time scales^58^, more comprehensive genomic sampling produced enhanced phylogenetic resolution of the Radula clade, where we focused the majority of phylogenetic comparative methods. In addition, we were unable to quantify the measurement error associated with the chemical traits within species, which can decrease the statistical power for detecting phylogenetic signal^56,59,60^. It is also possible that environmental effects on our chemical traits could bias estimates of phylogenetic signal and correlations^59^.

The causes of correlated evolution, including linkage, epistasis, and selection, are difficult to detect without careful approaches in quantitative genetics and population genomics. Nevertheless, one advantage of examining the presence/absence of multiple classes of defensive compounds in a phylogenetic context is that it is possible to test for expected patterns of correlated evolution due to shared metabolic pathways (e.g., flavonoids and cardenolides^7^) or due to adaptive advantages of specific mixtures. Recent studies detecting evolutionary associations among chemical traits^17,18^ have posited that the branching structure of metabolic pathways could potentially drive this pattern. If metabolite classes share a common precursor, one might expect evolutionary tradeoffs and negative covariation. Alternatively, if metabolite classes lie along the same metabolic pathway, an increase in one class may be concomitant with increases in another (or vice versa), causing positive covariation among the classes. There are also numerous empirical examples supporting the hypotheses that correlations may be driven by functional redundancy^61^ or selection for synergistic effects on herbivores^20^ rather than the structural constraints of metabolism. Suites of covarying defensive traits, or defense syndromes, have been detected in several plant genera^9,53^ and plant communities^62^, and have been predominantly used to describe covariation among mechanical and chemical defenses. It is interesting to note the correlated evolution of the flavones/chalcones and the *p*-alkenyl phenols/kavalactones could be due to metabolic constraints, as well as possible adaptations via synergistic (e.g., kavalactones in *P. methysticum*) or other mixture-associated defensive attributes^22^. Flavonoids and chalcones are directly linked biosynthetically, such that the inherent reactivity of the chalcone moiety permits the enzymatic processes that result in cyclization to the flavonoid scaffold (Fig. 3e). This strong biosynthetic tie yields a clear prediction that the presence of one would depend on the other, and indeed our structural analysis found many cases where both metabolite classes co-occurred in the same sample. Revealing the relationship between the kavalactones and *p*-alkenyl phenols is more tenuous because both classes are less prevalent across our samples. Kavalactones and *p*-alkenyl phenols are dramatically different compounds that diverge at a much earlier branch point from a common cinnamic/coumaric acid precursor. Whereas one polyacetate chain extension pathway leads to the long-chain lipophilic substituent, characteristic of the *p*-alkenyl phenols, the other chain extension pathway conserves oxidation states through the chain extension process to produce the lactones (kavalactones or butenolides) through cyclization reactions (Fig. 3e). The overall outcome is different than the chalcone-flavonoid relationship; in this case, two dramatically different compounds are produced by divergence from a common early-stage biosynthetic precursor in contrast to the immediate biosynthetic precursor relationship between chalcones and flavonoids. Broader sampling across *Piper* and Radula will be necessary to confirm this unexpected relationship between kavalactones and *p*-alkenyl phenols.

## Conclusion

Here we sought to advance understanding of phylogenetic relationships within *Piper* while simultaneously investigating the mode and manner of phytochemical evolution in this group. In addition to generating a well-resolved phylogeny, our results support theoretical expectations that broad classes of compounds display higher degrees of phylogenetic signal than molecular features revealed by ^1^H NMR data. In addition, trait associations observed in Radula can be used to pose functional hypotheses about genetic constraints or biases on phytochemical evolution and how these factors structure plant-animal interactions. Such investigations are one of the emerging frontiers in terrestrial ecology, and we hope that our study provides one example of how collaborative and multi-disciplinary research can progress in this area.

## Methods

### Study system and sample collection

For phylogenetic and chemical analyses, we collected leaf material from 71 individuals representing 65 Neotropical *Piper* species from the following clades: Churumayu (*N* = 3), Hemipodium (*N* = 1), Isophyllon (*N* = 5), Macrostachys (*N* = 4), Peltobryon (*N* = 2), Pothomorphe (*N* = 1), Radula (*N* = 44), and Schilleria (*N* = 5). This study complied with all local and national regulations/guidelines, and vouchers for all collections were deposited in herbaria in the country of origin as stipulated in the permit documents (Table S1). Brazilian collections were made under permit No. 15780-6 from the Sistema de Autorização e Informação em Biodiversidade (SISBIO). Costa Rican collections were made under the permits R-054-2018-OT-CONAGEBIO and R-055-2018-OT-CONAGEBIO from the Ministerio del Ambiente y Energía (MINAE). Collections from Ecuador were conducted under the permit 03-IC-FAU/FLO-DNP/MA granted by the Ministerio del Ambiente. Collections from Panamá were covered by the permit SE/AP-15-13 from the Autoridad Nacional del Ambiente (ANAM). Finally, Peruvian collections were covered by the permit 288-2015-SERFOR-DGGSPFFS granted by the Servicio Nacional Forestal de Fauna Silvestre (SERFOR). All collections were identified by E.J.T. in the field, and confirmed with vouchers in the herbarium using regional keys, where available, comparison with type specimens, and experience with the genus. For chemical profiling and DNA sequencing, we collected the youngest, fully expanded leaves and dried them immediately with silica gel. While drying on silica gel may not inhibit enzymatic activity and could limit our analyses to relatively stable molecules, this is not an issue for the phylogenetic analyses described below. Collections were only made from mature individuals in the field. Vouchers were pressed, dried, and deposited in one or more herbaria for future reference and species verification (Table S1). To investigate the evolution of phytochemistry at a relatively shallow evolutionary scale, we conducted the majority of our sampling within Radula^34^.

### Phylogenetic analyses

Genome-wide polymorphism data was generated for 71 individuals for phylogenetic analyses. Either the same accession sampled for chemical analysis, or an individual from the same population as the one sampled, were sequenced with a genotyping-by-sequencing approach^63^ that is analogous to ddRADseq^64^. Briefly, genomic DNA was digested with two restriction enzymes, *Eco*RI and *Mse*I. Sample-specific barcoded oligos containing Illumina adaptors were annealed to the *Eco*RI cut sites, and oligos containing the alternative Illumina adaptor were annealed to the *Mse*I cut sites. Fragments were PCR amplified and pooled for sequencing. The library was size-selected for fragments between 350 and 450 base pairs (bp) with the Pippin Prep System (Sage Sciences, Beverly, MA), and sequenced on two lanes of an Illumina HiSeq 2500 at the University of Texas Genome Sequencing and Analysis Facility (Austin, TX). Single-end, 100 bp, raw sequence data were filtered for contaminants (*E. coli*, *Phi*X, Illumina adaptors or primers) and low quality reads using bowtie2_db^65^ and a pipeline of bash and perl scripts (https://github.com/ncgr/tapioca). We used custom perl scripts to demultiplex our reads by individual and trim barcodes and restriction site-associated bases.

Assembly and initial filtering was conducted with ipyRAD v.0.7.30^66^. ipyRAD was specifically designed to assemble ddRADseq data for phylogenetic applications, permits customization of clustering and filtering, and allows for indel variation among samples^66^. Because a suitable *Piper* genome was not available at the time of analysis, we generated a de novo consensus reference of sampled genomic regions with ipyRAD. Briefly, nucleotide sites with phred quality scores lower than 33 were treated as missing data. Sequences were clustered within individuals according to an 85% similarity threshold with vsearch^67^ and aligned with muscle^68^ to produce stacks of highly similar ddRADseq reads (hereafter, ddRADseq loci). The sequencing error rate and heterozygosity were jointly estimated for all ddRADseq loci with a depth > 6, and these parameters informed statistical base calls according to a binomial model. Consensus sequences for each individual in the assembly were clustered once more, this time across individuals, and discarded if possessing > 8 indels (max_Indels_locus), > 50% heterozygous sites (max_shared_Hs_locus), or > 20% variable sites (max_SNPs_locus). To reduce the amount of missing data in our alignment matrix, ddRADseq loci were retained if they were present in at least 50 of 71 samples. The nexus file of concatenated consensus sequences for each individual, including invariant sites, was used as input for the Bayesian phylogenetic methods described below. Individual FASTQ files, nexus alignment, and complete information on additional parameter settings for this analysis are archived at Dryad (https://doi.org/10.5061/dryad.j6q573nc7).

To resolve patterns of diversification and to provide a foundation for investigating variation in patterns of phytochemical evolution, we estimated a rooted, calibrated tree according to a relaxed clock model in RevBayes v.1.0.12^69^, which provides the ability to specify custom phylogenetic models for improved flexibility compared with other Bayesian approaches. The prior distribution on node ages was defined by a birth–death process in which the hyper priors on speciation and extinction rates were exponentially distributed with *λ* = 10. We relaxed the assumption of a global molecular clock by allowing each branch-rate variable to be drawn from a lognormal distribution. After comparing the relative fits of JC, HKY, GTR, and GTR + Gamma nucleotide substitution models with Bayes factors, we modeled DNA sequence evolution according to the best-fit HKY model. Eight independent MCMC chains were run for 100,000 generations with a burn-in of 1,000 generations and sampled every 10 generations. Chains were visually assessed for convergence with Tracer v.1.7.1^70^ and numerically assessed with effective sample sizes (ESS), the Gelman − Rubin convergence diagnostic^71^, and by comparing the posterior probabilities of clades sampled between MCMC chains. The maximum clade credibility (MCC) tree provided the ultrametric fixed tree topology and relative node ages for phylogenetic comparative methods described below.

### Chemical profiling

Crude proton nuclear magnetic resonance (^1^H NMR) spectroscopy was chosen for chemotype mapping due to its ability to characterize subtle structural variation across a wide range of compound classes in a single, reproducible, non-destructive analysis^39^. Briefly, after leaf samples were ground to fine powder, approximately 100.0–2000.0 mg of leaf material were ground and transferred to a glass screw cap test tube with 10 ml of methanol, sonicated for 10 min, and filtered. This step was repeated and both filtrates were combined in a pre-weighed 20 ml scintillation vial. The solvent was removed *in vacuo* and dissolved in 0.6 ml methanol-*d*_4_ for ^1^H NMR analysis. Crude ^1^H NMR solutions were standardized to 13.1 ± 3.8 mg/mL when possible and analyzed on a Varian 400 MHz solution state NMR spectrometer with autosampler. Data were processed using MestReNova software (Mestrelab Research, Santiago de Compostela, Spain). Spectra from the crude extracts were aligned with the solvent peak (CD_3_, δ = 3.31 ppm), baseline corrected, and phase corrected. Solvent and water peaks were removed and the binned spectra were normalized to a total area of 100. This data set is referred to as “crude ^1^H NMR”.

In addition to crude ^1^H NMR spectral chemotyping, we further annotated samples based upon the presence or absence of compound classes. To further gain structural resolution across the crude extracts that were sampled, aliquots of the ^1^H NMR extracts were diluted and subjected to GC–MS and LC–MS analysis (see Supplementary Information for additional details). Crude extracts were classified using chemotaxonomic classifications outlined in Parmar’s comprehensive review of *Piper* phytochemistry^35^, and our rationale for assigning chemical classes is outlined for each species in Table S2. Briefly, phenolic compounds were identified from high-resolution matches to the METLIN mass spectrometry database^72^. Database hits were then confirmed by agreement of crude ^1^H NMR chemical shifts with literature values for phenolics known to be found in *Piper*, but not always Radula species. Many compounds identified by LC–MS as flavonoids and chalcones had multiple possible METLIN matches, which confounded NMR confirmation. In these cases, we were still able to differentiate flavonoids from chalcones by characteristic UV spectra (l_max_ ~ 350 nm). Phenylpropanoids and *p-*alkenyl phenols were identified based on characteristic GC–MS fragmentation for these compound classes known to be found in *Piper*. *Piper* amides were characterized in a similar fashion, starting from high-resolution mass spectrometric matches and confirming with known ^1^H NMR data from the literature. In some cases, crude 2D-NMR analysis (COSY, HSQC) was used to confirm structural classifications. COrrelated SpectroscopY (COSY) was used to identify ^1^H NMR that were contained within the same molecule, while Heteronuclear Single Quantum Coherence (HSQC) spectroscopy was used to identify the carbon (^13^C) resonances associated with certain proton (^1^H) signals to verify the presence of specific functional groups^73^. Only the most abundant and spectroscopically apparent compounds were classified due to the low sensitivity of NMR. 35 total classes were identified at three levels of structural resolution. At the coarsest level of resolution, we identified compounds as phenolics, nitrogen-containing, or sesquiterpenes. Within the phenolics, we identified nine intermediate and 17 high-resolution subclasses. Within the nitrogen-containing compounds, we identified three intermediate and three high-resolution subclasses. Finer resolution was not characterized for the sesquiterpene class. This hierarchical set of 35 traits is referred to as “metabolite classes” (Fig. 2). Additional details on chemical profiling can be found in the Supplementary Information.

### Phylogenetic signal and evolution of metabolite classes

To assess whether metabolite classes were phylogenetically conserved across Radula, we quantified phylogenetic signal in these binary traits using the D statistic^57^. The D statistic calculates the sum of sister-clade differences, Σd_obs_ for an observed tree and binary trait, and scales this value with the distributions of sums expected under two disparate evolutionary models, random and Brownian motion (Σd_r_ and Σd_b_, respectively), using the following equation:

$$D=\frac{\left[\Sigma {d}_{obs}-mean\left(\Sigma {d}_{b}\right)\right]}{\left[mean\left(\Sigma {d}_{r}\right)- mean\left(\Sigma {d}_{b}\right)\right]}$$

Thus, D is expected to equal 1 when the observed binary trait is distributed randomly, lacking phylogenetic signal, and is expected to equal 0 when it exhibits phylogenetic signal as expected under Brownian motion. As tests of phylogenetic signal with the D statistic are most accurate when the ratio of presences and absences is closer to 1:1^57^, we tested for phylogenetic signal in eight of the 35 metabolite classes (outlined in white in Fig. 2) which were present in a sufficient proportion of taxa. We used the *phylo.d* function in the caper package^74^ in R v.4.0.0^75^ to calculate the observed D for a subset of binary traits that were sufficiently present across the phylogeny. This value was compared to a distribution of D values simulated under models of phylogenetic randomness (D = 1) and pure Brownian motion (D = 0) to determine whether the observed D differed from either zero or one.

To detect evolutionary associations among pairs of metabolite classes within Radula, we used Pagel’s method^76^ that models evolutionary changes in two binary traits, X and Y, as continuous-time Markov processes in which the probabilities of state transition at one trait may depend on the state at the other trait. We tested all pairwise associations among the eight metabolite classes that were represented by a sufficient number of Radula taxa to provide accurate tests of evolutionary associations (*N* = 28). Significant tests of correlated evolution were followed by tests of contingency, in which changes at X depend on the state of Y, or vice versa. Model fits, comparisons, and plots were performed with the *fitPagel* function in the phytools package^77^ in R.

### Multivariate analyses of phylogenetic signal with crude ^1^H NMR spectra

While the analyses above based on broad classifications of structurally determined metabolites provide a coarse view of phytochemical evolution, these classifications are anchored to the foundations of plant secondary metabolite biosynthesis. Using ^1^H NMR spectra as a raw chemotype should allow a more detailed multivariate perspective on phytochemical diversity. Studies on other plant taxa have typically detected some signal and evolutionary correlations for broad classes of compounds but not necessarily for specific compounds or biologically active moieties, both of which can be inferred from ^1^H NMR data. Multivariate approaches to phylogenetic comparative methods have provided insight into covarying suites of related traits, while simultaneously increasing the statistical power to detect phylogenetic signal^78^ and differences in trait means among taxa^79^. Indeed, these multivariate approaches might be particularly useful when exploring the evolution of complex phenotypes, like the plant metabolome, which exhibit trait covariances due to metabolomic or functional associations^20^. Here we utilize three multivariate methods to detect patterns of phylogenetic signal for 263 resonances found in the crude ^1^H NMR data representing all 35 metabolite classes: (1) principal coordinate analyses (PCoA); (2) multiple regression on distance matrices (MRM); and (3) multivariate estimation of phylogenetic signal.

To visualize patterns of chemotypic variation across all sampled species from all clades, we first analyzed the ^1^H NMR data with PCoA. First we calculated the Manhattan distances between all pairwise species with the *dist* function in R, and then conducted PCoA on the distance matrix using the *pcoa* function in R. If the major axes of metabolomic variation are phylogenetically conserved, the plotted species scores should be clustered by clade in a rotated principal coordinate (PCo) space. Alternatively, if metabolomic variation is randomly distributed across the phylogeny, there should be little to no clustering by clade^80^. The degree to which plant clade predicted chemical similarity was assessed using permutational multivariate analysis of variance (permanova)^81^ in the vegan package^82^ in R based on Euclidean distances of the first four PCo axes.

Mantel tests have been frequently used to assess the degree of phylogenetic signal in multivariate data^10,83,84^ by estimating the relationship between phylogenetic and phenotypic distances. Simulations under scenarios of measurement error have found instances where Mantel tests outperform traditional univariate methods in detecting phylogenetic signal, especially as the number of traits increases^60^. Because we were unable to account for measurement error in our study, we utilized Multiple Regression on distance Matrices (MRM)^85^ to examine the relationship between metabolomic and phylogenetic distance at two evolutionary scales (within Radula and across all clades). Euclidean distances were calculated from the crude ^1^H NMR spectra using the *dist* function in R, and phylogenetic distances for Radula only and all clades were calculated using the *cophenetic.phylo* function in the ape package^86^ in R. MRM analyses were implemented using the *MRM* function with 1000 permutations in the ecodist package^87^ in R.

Since Blomberg’s *K*^56^ statistic exhibits higher statistical power to detect phylogenetic signal relative to Mantel tests^88^, we quantified phylogenetic signal of the crude ^1^H NMR at both evolutionary scales using a multivariate generalization of the *K* statistic (*K*_mult_)^89^ with the *physignal* function in the geomorph package^90^ in R. Similar to the aforementioned D statistic, the K statistic compares the observed variation to that expected under Brownian motion, but the K statistic does not scale this comparison by the variation exhibited under a completely random evolutionary model^56,89^. Values of *K* greater than 1 indicate phylogenetic signal greater than expected under Brownian motion, whereas values between 0 and 1 indicate less signal than expected under Brownian motion. Significance for the generalized *K* statistic was assessed by permuting the ^1^H NMR peak data among the tips of the phylogeny for 999 iterations. To determine whether the zero-inflated nature of the ^1^H NMR data influenced the detection of phylogenetic signal, we permuted our ^1^H NMR data set over 1000 iterations by randomly indexing our original ^1^H NMR data matrix. This permutation method preserves the original proportion of zeros in the matrix while obfuscating any observed phylogenetic signal. The generalized *K* statistic test was calculated for each permutation, and our observed generalized *K* statistic was compared to the null distribution of permuted values.

## Supplementary Information

Supplementary Information.

## References

[1] Thompson JN, Pellmyr O (1991). Evolution of oviposition behavior and host preference in Lepidoptera. Annu. Rev. Entomol..

[2] Bowers MD (1984). Iridoid glycosides and host-plant specificity in larvae of the buckeye butterfly, *Junonia coenia* (Nymphalidae). J. Chem. Ecol..

[3] Zagrobelny M (2004). Cyanogenic glucosides and plant–insect interactions. Phytochemistry.

[4] Richards LA (2012). Synergistic effects of iridoid glycosides on the survival, development and immune response of a specialist caterpillar, *Junonia coenia* (Nymphalidae). J. Chem. Ecol..

[5] Berenbaum M (1978). Toxicity of a furanocoumarin to armyworms: A case of biosynthetic escape from insect herbivores. Science.

[6] Ehrlich PR, Raven PH (1964). Butterflies and plants: A study in coevolution. Evolution.

[7] Agrawal AA, Salminen JP, Fishbein M (2009). Phylogenetic trends in phenolic metabolism of milkweeds (*Asclepias*): Evidence for escalation. Evolution.

[8] Maron JL, Agrawal AA, Schemske DW (2019). Plant-herbivore coevolution and plant speciation. Ecology.

[9] Agrawal AA, Fishbein M (2006). Plant defense syndromes. Ecology.

[10] Salazar D (2018). Origin and maintenance of chemical diversity in a species-rich tropical tree lineage. Nat. Ecol. Evol..

[11] Griffin WJ, Lin GD (2000). Chemotaxonomy and geographical distribution of tropane alkaloids. Phytochemistry.

[12] Wink M (2003). Evolution of secondary metabolites from an ecological and molecular phylogenetic perspective. Phytochemistry.

[13] Zhang Y (2020). Phylogenetic patterns suggest frequent multiple origins of secondary metabolites across the seed plant “tree of life”. Natl. Sci. Rev..

[14] Kursar TA (2009). The evolution of antiherbivore defenses and their contribution to species coexistence in the tropical tree genus *Inga*. Proc. Natl. Acad. Sci. USA.

[15] Salazar D, Jaramillo MA, Marquis RJ (2016). Chemical similarity and local community assembly in the species rich tropical genus *Piper*. Ecology.

[16] Allevato DM, Groppo M, Kiyota E, Mazzafera P, Nixon KC (2019). Evolution of phytochemical diversity in *Pilocarpus* (Rutaceae). Phytochemistry.

[17] Boachon B (2018). Phylogenomic mining of the mints reveals multiple mechanisms contributing to the evolution of chemical diversity in Lamiaceae. Mol. Plant.

[18] Johnson MT, Ives AR, Ahern J, Salminen JP (2014). Macroevolution of plant defenses against herbivores in the evening primroses. New Phytol..

[19] Agrawal AA (2007). Macroevolution of plant defense strategies. Trends Ecol. Evol..

[20] Richards LA, Dyer LA, Smilanich AM, Dodson CD (2010). Synergistic effects of amides from two *Piper* species on generalist and specialist herbivores. J. Chem. Ecol..

[21] Sedio BE (2017). Recent breakthroughs in metabolomics promise to reveal the cryptic chemical traits that mediate plant community composition, character evolution and lineage diversification. New Phytol..

[22] Dyer LA (2018). Modern approaches to study plant–insect interactions in chemical ecology. Nat. Rev. Chem..

[23] Richards LA (2016). Phytochemical diversity and synergistic effects on herbivores. Phytochem. Rev..

[24] Sedio BE, Parker JD, McMahon SM, Wright SJ (2018). Comparative foliar metabolomics of a tropical and a temperate forest community. Ecology.

[25] Fine PVA (2006). The growth–defense trade-off and habitat specialization by plants in Amazonian forests. Ecology.

[26] Léveillé-Bourret É, Chen BH, Garon-Labrecque MÉ, Ford BA, Starr JR (2020). RAD sequencing resolves the phylogeny, taxonomy and biogeography of Trichophoreae despite a recent rapid radiation (Cyperaceae). Mol. Phylogenet. Evol..

[27] Parchman TL, Jahner JP, Uckele KA, Galland LM, Eckert AJ (2018). RADseq approaches and applications for forest tree genetics. Tree Genet. Genomes.

[28] Massatti R, Reznicek AA, Knowles LL (2016). Utilizing RADseq data for phylogenetic analysis of challenging taxonomic groups: A case study in *Carex* sect. *Racemosae*. Am. J. Bot..

[29] Du ZY, Harris AJ, Xiang QYJ (2020). Phylogenomics, co-evolution of ecological niche and morphology, and historical biogeography of buckeyes, horsechestnuts, and their relatives (Hippocastaneae, Sapindaceae) and the value of RAD-seq for deep evolutionary inferences back to the Late Cretaceous. Mol. Phylogenet. Evol..

[30] Fernández-Mazuecos M (2017). Resolving recent plant radiations: power and robustness of genotyping-by-sequencing. Syst. Biol..

[31] Paetzold C, Wood KR, Eaton D, Wagner WL, Appelhans MS (2019). Phylogeny of Hawaiian *Melicope* (Rutaceae): RAD-Seq resolves species relationships and reveals ancient introgression. Front. Plant Sci..

[32] Eaton DA, Spriggs EL, Park B, Donoghue MJ (2017). Misconceptions on missing data in RAD-seq phylogenetics with a deep-scale example from flowering plants. Syst. Biol..

[33] Callejas-Posada, R. Piperaceae. in *Flora Mesoamericana* Vol. 2, pt. 2 (eds. Davidse, G., Ulloa Ulloa, C., Hernández, H. M. & Knapp, S.) 1–618 (Missouri Botanical Garden Press, 2020).

[34] Martínez C, Carvalho MR, Madriñán S, Jaramillo CA (2015). A late Cretaceous *Piper* (Piperaceae) from Colombia and diversification patterns for the genus. Am. J. Bot..

[35] Parmar VS (1997). Phytochemistry of the genus *Piper*. Phytochemistry.

[36] Dyer, L. A. & Palmer, A. D. N. *Piper: A Model Genus for Studies of Phytochemistry, Ecology, and Evolution*. (Kluwer Academic/Plenum Publishers, 2004).

[37] Richards LA (2015). Phytochemical diversity drives plant–insect community diversity. Proc. Natl. Acad. Sci. USA.

[38] Kato MJ, Furlan M (2007). Chemistry and evolution of the Piperaceae. Pure Appl. Chem..

[39] Richards LA, Oliveira C, Dyer LA (2018). Shedding light on chemically mediated tri-trophic interactions: A ^1^H-NMR network approach to identify compound structural features and associated biological activity. Front. Plant Sci..

[40] Jahner JP (2017). Host conservatism, geography, and elevation in the evolution of a Neotropical moth radiation. Evolution.

[41] Glassmire AE (2016). Intraspecific phytochemical variation shapes community and population structure for specialist caterpillars. New Phytol..

[42] Smith JF, Stevens AC, Tepe EJ, Davidson C (2008). Placing the origin of two species-rich genera in the late cretaceous with later species divergence in the tertiary: a phylogenetic, biogeographic and molecular dating analysis of *Piper* and *Peperomia* (Piperaceae). Plant Syst. Evol..

[43] Jaramillo MA (2008). A phylogeny of the tropical genus *Piper* using ITS and the chloroplast intron psbJ–petA. Syst. Bot..

[44] Molina-Henao YF, Guerrero-Chacón AL, Jaramillo MA (2016). Ecological and geographic dimensions of diversification in *Piper* subgenus *Ottonia*: A lineage of Neotropical rainforest shrubs. Syst. Bot..

[45] Asmarayani R (2018). Phylogenetic relationships in Malesian-Pacific *Piper* (Piperaceae) and their implications for systematics. Taxon.

[46] Salehi B (2019). *Piper* species: A comprehensive review on their phytochemistry, biological activities and applications. Molecules.

[47] Cariou M, Duret L, Charlat S (2013). Is RAD-seq suitable for phylogenetic inference? An in silico assessment and optimization. Ecol. Evol..

[48] Yonekura-Sakakibara K, Higashi Y, Nakabayashi R (2019). The origin and evolution of plant flavonoid metabolism. Front. Plant Sci..

[49] Freitas GC (2014). Cytotoxic non-aromatic B-ring flavanones from *Piper carniconnectivum* C. DC. Phytochemistry.

[50] Hunyadi A, Martins A, Danko B, Chang FR, Wu YC (2014). Protoflavones: A class of unusual flavonoids as promising novel anticancer agents. Phytochem. Rev..

[51] Latif AD (2020). Protoflavone-chalcone hybrids exhibit enhanced antitumor action through modulating redox balance, depolarizing the mitochondrial membrane, and inhibiting ATR-dependent signaling. Antioxidants.

[52] Revell LJ, Harmon LJ, Collar DC (2008). Phylogenetic signal, evolutionary process, and rate. Syst. Biol..

[53] Endara MJ (2017). Coevolutionary arms race versus host defense chase in a tropical herbivore-plant system. Proc. Natl. Acad. Sci. USA.

[54] Kamilar JM, Cooper N (2013). Phylogenetic signal in primate behaviour, ecology and life history. Philos. Trans. R. Soc. B.

[55] Garamszegi LZ, Møller AP (2011). Nonrandom variation in within-species sample size and missing data in phylogenetic comparative studies. Syst. Biol..

[56] Blomberg SP, Garland T, Ives AR (2003). Testing for phylogenetic signal in comparative data: behavioral traits are more labile. Evolution.

[57] Fritz SA, Purvis A (2010). Selectivity in mammalian extinction risk and threat types: A new measure of phylogenetic signal strength in binary traits. Conserv. Biol..

[58] Sakamoto M, Venditti C (2018). Phylogenetic non-independence in rates of trait evolution. Biol. Lett..

[59] Ives AR, Midford PE, Garland T (2007). Within-species variation and measurement error in phylogenetic comparative methods. Syst. Biol..

[60] Hardy OJ, Pavoine S (2012). Assessing phylogenetic signal with measurement error: A comparison of Mantel tests, Blomberg et al.’s K, and phylogenetic distograms. Evolution.

[61] Romeo, J. T., Saunders, J. A. & Barbosa, P. *Phytochemical Diversity and Redundancy in Ecological Interactions*, Vol. 30. (Springer, 2013).

[62] Kursar TA, Coley PD (2003). Convergence in defense syndromes of young leaves in tropical rainforests. Biochem. Syst. Ecol..

[63] Parchman TL (2012). Genome-wide association genetics of an adaptive trait in lodgepole pine. Mol. Ecol..

[64] Peterson BK, Weber JN, Kay EH, Fisher HS, Hoekstra HE (2012). Double digest RADseq: An inexpensive method for *de novo* SNP discovery and genotyping in model and non-model species. PLoS ONE.

[65] Langmead B, Salzberg SL (2012). Fast gapped-read alignment with Bowtie 2. Nat. Methods.

[66] Eaton DA (2014). PyRAD: Assembly of *de novo* RADseq loci for phylogenetic analyses. Bioinformatics.

[67] Rognes T, Flouri T, Nichols B, Quince C, Mahé F (2016). VSEARCH: A versatile open source tool for metagenomics. PeerJ.

[68] Edgar RC (2004). MUSCLE: Multiple sequence alignment with high accuracy and high throughput. Nucleic Acids Res..

[69] Höhna S (2016). RevBayes: Bayesian phylogenetic inference using graphical models and an interactive model-specification language. Syst. Biol..

[70] Rambaut A, Drummond AJ, Xie D, Baele G, Suchard MA (2018). Posterior summarization in Bayesian phylogenetics using Tracer 1.7. Syst. Biol..

[71] Gelman A, Rubin DB (1992). Inference from iterative simulation using multiple sequences. Statist. Sci..

[72] Guijas C (2018). METLIN: a technology platform for identifying knowns and unknowns. Anal. Chem..

[73] Crews P, Rodríguez J, Jaspars M (2010). Organic Structure Analysis.

[74] Orme, D. *et al.* caper: Comparative analyses of phylogenetics and evolution in R. R package version 1.0.1. https://CRAN.R-project.org/package=caper (2018)

[75] R Core Team. *R: A Language and Environment for Statistical Computing*. (R Foundation for Statistical Computing, https://www.R-project.org/, 2020).

[76] Pagel M (1994). Detecting correlated evolution on phylogenies: A general method for the comparative analysis of discrete characters. Proc. R. Soc. B.

[77] Revell LJ (2012). phytools: An R package for phylogenetic comparative biology (and other things). Methods Ecol. Evol..

[78] Zheng L (2009). New multivariate tests for phylogenetic signal and trait correlations applied to ecophysiological phenotypes of nine *Manglietia* species. Funct. Ecol..

[79] Clavel J, Escarguel G, Merceron G (2015). mvmorph: An R package for fitting multivariate evolutionary models to morphometric data. Methods Ecol. Evol..

[80] Klingenberg CP, Gidaszewski NA (2010). Testing and quantifying phylogenetic signals and homoplasy in morphometric data. Syst. Biol..

[81] Anderson MJ (2001). A new method for non-parametric multivariate analysis of variance. Austral Ecol..

[82] Oksanen, J. *et al.* vegan: Community Ecology Package, R package version 2.5-7. https://CRAN.R-project.org/package=vegan (2020)

[83] Cardini A, Elton S (2008). Does the skull carry a phylogenetic signal? Evolution and modularity in the guenons. Biol. J. Linn. Soc..

[84] Easson CG, Thacker RW (2014). Phylogenetic signal in the community structure of host-specific microbiomes of tropical marine sponges. Front. Microbiol..

[85] Lichstein JW (2007). Multiple regression on distance matrices: A multivariate spatial analysis tool. Plant Ecol..

[86] Paradis E, Claude J, Strimmer K (2004). APE: Analyses of phylogenetics and evolution in R language. Bioinformatics.

[87] Goslee SC, Urban DL (2007). The ecodist package for dissimilarity-based analysis of ecological data. J. Stat. Softw..

[88] Harmon LJ, Glor RE (2010). Poor statistical performance of the Mantel test in phylogenetic comparative analyses. Evolution.

[89] Adams DC (2014). A generalized *K* statistic for estimating phylogenetic signal from shape and other high-dimensional multivariate data. Syst. Biol..

[90] Adams DC, Otárola-Castillo E (2013). geomorph: An R package for the collection and analysis of geometric morphometric shape data. Methods Ecol. Evol..

