# Supplementary material for: Phytochemistry reflects different evolutionary history in traditional classes versus specialized structural motifs
Source: Sci Rep. 2021 Aug 26;11:17247. doi: 10.1038/s41598-021-96431-3 (PMC8390663; doi:10.1038/s41598-021-96431-3)
Supplement: Supplementary file 1 — Supplementary Information. [file 41598_2021_96431_MOESM1_ESM.pdf]

Supplementary Information

Phytochemistry reflects different evolutionary history in traditional classes versus specialized structural motifs

Kathryn A. Uckele, Joshua P. Jahner, Eric J. Tepe, Lora A. Richards, Lee A. Dyer, Kaitlin M. Ochsenrider, Casey S. Philbin, Massuo J. Kato, Lydia F. Yamaguchi, Matthew L. Forister, Angela M. Smilanich, Craig D. Dodson, Christopher S. Jeffrey, Thomas L. Parchman

Supplementary Methods

Nuclear magnetic resonance spectroscopy

Foliar tissue (100.0-2000.0 mg) from each sample was cooled with liquid nitrogen and ground to a fine powder using a mortar and pestle and was combined with 10 mL of methanol (HPLC grade, Fisher Scientific, Pittsburgh, PA). Samples were vortexed for 30 seconds, sonicated for 10 minutes before the supernatant was decanted and syringe filtered (0.45 µm). This process was repeated once on the retained plant material and filtered supernatants were combined and concentrated to dryness using a Genevac centrifugal evaporator. Samples were placed on a hi-vac overnight before determining extract mass. All samples underwent a deuterium exchange (HDX) to minimize proton peaks in ^{1}H NMR spectra by thrice reconstituting in methanol-d1 (Cambridge Isotope Laboratories, Tewksbury, MA) and re-drying. In cases where extract mass was above 20 mg, extracts were aliquoted to standardize extract mass to 13.1±3.8 mg/mL, and in some cases extract mass was below this mean (2.0-17.9 mg). Dried deuterated extracts were reconstituted in 600 µL methanol-d4 with 0.01% TMS and syringe filtered into NMR tubes for ^{1}H NMR analysis. FIDs were collected for each sample on a Varian 400 MHz NMR spectrometer (128 scans) before processing using MestReNova software (Mestrelab Research, Spain). Spectra were aligned to the residual methanol solvent peak ($C\underline{D}_3OD$, 3.31 ppm), Global phase-corrected, Whittaker baseline-corrected, and peak picked before being used for structural annotation. Further isolation was completed on crude extracts of *P. holdridgeanum, P. cabagranum,* and *P. peracuminatum* using a combination of flash column chromatography and reverse phase C18 preparatory medium pressure chromatography. Structural elucidation was completed on these isolated compounds using ^{1}H, ^{13}C, COSY, HSQC, NOESY, and HMBC NMR spectroscopy.

Liquid and gas chromatography-mass spectrometry

During the first step of HDX, 100 uL of ^{1}H NMR stock was was diluted 1:10 with protonated methanol for LC-MS analysis. Extracts were injected (1 µL) onto an Agilent (Santa Clara, CA) 1200 analytical HPLC equipped with a binary pump, autosampler, column compartment and diode array UV detector and eluted at 0.500 mL/min through a Kinetex EVO C18 column (Phenomenex, 2.1 x 100 mm, 2.6 µ, 100 Å; Torrance, CA) at 40 °C. The linear binary gradient was comprised of buffers A (Optima-grade water containing 0.1 % formic acid, Fisher Scientific) and B (Optima-grade acetonitrile containing 0.1 % formic acid, Fisher Scientific) changing over 20 minutes accordingly: 0-1 min 20% B, ramp to 50% B at 6 min, ramp to 100% B at 12 min, 12-16 min hold at 100% B, 16-17 min ramp to 20% B, 17-20 min hold at 20% B. Liquid chromatography was coupled to an Agilent 6230 Time-of-Flight mass spectrometer via an electrospray ionization source (ESI-TOF; gas temperature: 325 °C, flow: 10 L/m; nebulizer pressure: 35 psig; VCap: 3500 V; fragmentor: 165 V; skimmer: 65 V; octopole: 750 V). Raw data were processed and analysed in Agilent MassHunter.

A portion of the crude extract (~ 1-3 mg) was dissolved in CH_2Cl_2 (2 mL) and analyzed in the UNR Chemistry Department Shared Instrument Lab using an HP Agilent 7890A GC System coupled with an Agilent 5975C MSD (Agilent Technologies, Santa Clara, CA, USA), equipped with a DB-Ultra Inert capillary column (30 m x 250 µm x 0.25 µm; Agilent J&W GC Columns, Santa Clara, CA, USA). The carrier gas used was ultra-pure He set at a flow rate of 1.0

mL/min with a pressure of XX psi with an injection port temperature of 250 °C. Initial oven temperature 80 °C, with an initial hold time of 1.5 min, then ramping at 17°C/min to 300 °C, with a final hold time of 15 min. The resulting GC-MS data were recorded and processed using MassHunter Workstation Quantitative Analysis software. Comparisons to literature values and NIST database searches was used to complete the categorical analysis, which is detailed in the supplementary table S2.

Table S1. Sampling information for all taxa. Herbarium acronyms are as follows: CINC=Margaret H. Fulford Herbarium, University of Cincinnati; CR=Herbario Nacional at the Museo Nacional de Costa Rica, San José, Costa Rica; PMA=Herbarium at the Universidad de Panamá, Panamá; QCNE=Herbario Nacional del Ecuador at the Museo Ecuatoriano de Ciencias Naturales del Instituto Nacional de Biodiversidad, Quito, Ecuador; SPF=Herbarium of the Universidade de São Paulo, São Paulo, Brazil; USM=Herbarium of the Universidad Nacional de San Marcos, Lima, Perú.

Species	Country	Collector	Voucher (herbarium)	Clade
P. aduncum var. *cordulatum* (C.DC.) Yunck.	Brazil	M. Kato	*M. Kato K-1978 (SPF)*	Radula
P. arcteacuminatum Trel.	Costa Rica	E.J. Tepe	*E.J. Tepe 3534 (CR)*	Radula
P. amphioxys Trel.	Panamá	E.J. Tepe	*E.J. Tepe 4044 (PMA)*	Schilleria
P. armatum Trel. & Yunck.	Peru	E.J. Tepe	*E.J. Tepe 4394 (USM)*	Radula
P. baezense Trel.	Ecuador	A.E. Glassmire	*A.E. Glassmire YY1 (CINC)*	Radula
P. barbatum Kunth	Ecuador	E.J. Tepe	*E.J. Tepe 3005 (QCNE)*	Churumayu
P. cabagranum C.DC.	Costa Rica	E.J. Tepe	*E.J. Tepe 3531 (CR)*	Schilleria
P. carrilloanum C.DC.	Panamá	E.J. Tepe	*E.J. Tepe 4069 (PMA)*	Schilleria
P. cenocladum C.DC.	Panamá	E.J. Tepe	*E.J. Tepe 3999 (PMA)*	Macrostachys
P. chanchamayanum Trel.	Peru	E.J. Tepe	*E.J. Tepe 4393 (USM)*	Radula
P. changuinolanum Trel.	Panamá	E.J. Tepe	*E.J. Tepe 3961 (PMA)*	Radula
P. chimonanthifolium Kunth	Brazil	M. Kato	*M. Kato K-1960 (SPF)*	Radula
P. chrysostachyum C.DC.	Costa Rica	E.J. Tepe	*E.J. Tepe 3482 (CR)*	Radula
P. colonense C.DC. (1)	Panamá	E.J. Tepe	*E.J. Tepe 4032 (PMA)*	Radula
P. colonense C.DC. (2)	Costa Rica	E.J. Tepe	*E.J. Tepe 3502 (CR)*	Radula
P. crassinervium Kunth (1)	Costa Rica	E.J. Tepe	*E.J. Tepe 3463 (CR)*	Churumayu
P. crassinervium Kunth (2)	Brazil	M. Kato	*M. Kato K-1954 (SPF)*	Churumayu
P. culebranum C.DC. (1)	Costa Rica	E.J. Tepe	*E.J. Tepe 3413 (CR)*	Radula
P. culebranum C.DC. (2)	Costa Rica	E.J. Tepe	*E.J. Tepe 3527 (CR)*	Radula
P. culebranum C.DC. (3)	Panamá	E.J. Tepe	*E.J. Tepe 4005 (PMA)*	Radula
P. cyanophyllum Trel.	Costa Rica	E.J. Tepe	*E.J. Tepe 3427 (CR)*	Peltobryon
P. cyphophyllum C.DC.	Costa Rica	E.J. Tepe	*E.J. Tepe 3541 (CR)*	Radula
P. disparipes Trel. (1)	Costa Rica	E.J. Tepe	*E.J. Tepe 3426 (CR)*	Radula
P. disparipes Trel. (2)	Costa Rica	E.J. Tepe	*E.J. Tepe 3543 (CR)*	Radula
P. disparipes Trel. (3)	Costa Rica	E.J. Tepe	*E.J. Tepe 3486 (CR)*	Radula
P. distigmatum Yunck.	Panamá	E.J. Tepe	*E.J. Tepe 3972 (PMA)*	Isophyllon
P. dryadanum C.DC.	Panamá	E.J. Tepe	*E.J. Tepe 3963 (PMA)*	Radula
P. euryphyllum C.DC.	Panamá	E.J. Tepe	*E.J. Tepe 4000 (PMA)*	Macrostachys
P. figlinum Trel.	Costa Rica	E.J. Tepe	*E.J. Tepe 3483 (CR)*	Isophyllon
P. fimbriulatum C.DC.	Panamá	E.J. Tepe	*E.J. Tepe 3956 (PMA)*	Macrostachys
P. friedrichsthalii C.DC.	Costa Rica	E.J. Tepe	*E.J. Tepe 131 (CR)*	Radula
P. gaudichaudianum Kunth (1)	Brazil	M. Kato	*M. Kato K-1949 (SPF)*	Radula
P. gaudichaudianum Kunth (2)	Brazil	M. Kato	*M. Kato K-1983 (SPF)*	Radula
P. goesii Yunck.	Brazil	M. Kato	*M. Kato K-1964 (SPF)*	Schilleria
P. gonocarpum Trel.	Panamá	E.J. Tepe	*E.J. Tepe 3959 (PMA)*	Isophyllon
P. hartwegianum (Benth.) C.DC.	Panamá	E.J. Tepe	*E.J. Tepe 3966 (PMA)*	Macrostachys
P. hispidum Sw. (1)	Costa Rica	E.J. Tepe	*E.J. Tepe 3430 (CR)*	Radula
P. hispidum s.l. (2)	Costa Rica	E.J. Tepe	*E.J. Tepe 3476 (CR)*	Radula

P. hispidum Sw. (3)	Costa Rica	E.J. Tepe	*E.J. Tepe 3496 (CR)*	Radule
P. hispidum Sw. (4)	Costa Rica	E.J. Tepe	*E.J. Tepe 3509 (CR)*	Radula
P. hispidum Sw. (5)	Costa Rica	E.J. Tepe	*E.J. Tepe 3537 (CR)*	Radula
P. holdridgeanum W.C.Burger	Costa Rica	E.J. Tepe	*E.J. Tepe 4196 (CR)*	Unclassified
P. lagoense C.DC.	Brazil	M. Kato	*M. Kato K-1944 (SPF)*	Radula
P. latibracteum C.DC.	Panamá	E.J. Tepe	*E.J. Tepe 4050 (PMA)*	Isophyllon
P. longicaudatum Trel. & Yunck.	Ecuador	E.J. Tepe	*E.J. Tepe 3036 (QCNE)*	Radula
P. lucigaudens C.DC. (1)	Panamá	E.J. Tepe	*E.J. Tepe 3993 (PMA)*	Radula
P. lucigaudens C.DC. (2)	Panamá	E.J. Tepe	*E.J. Tepe 4028 (PMA)*	Radula
P. malacophyllum (C.Presl.) C.DC.	Brazil	M.Kato	*M. Kato K-1945 (SPF)*	Radula
P. maranyonense Trel.	Ecuador	E.J. Tepe	*E.J. Tepe 3034 (QCNE)*	Peltobryon
P. mollicomum Kunth	Brazil	M. Kato	*M. Kato K-1942 (SPF)*	Radula
P. mosenii C.DC.	Brazil	M. Kato	*M. Kato 1948 (SPF)*	Radula
P. peracuminatum C.DC. (1)	Costa Rica	E.J. Tepe	*E.J. Tepe 3433 (CR)*	Radula
P. peracuminatum C.DC. (2)	Panamá	E.J. Tepe	*E.J. Tepe 4062 (PMA)*	Radula
P. persubulatum C.DC.	Panamá	E.J. Tepe	*E.J. Tepe 4068 (PMA)*	Radula
P. polytrichum C.DC. (1)	Panamá	E.J. Tepe	*E.J. Tepe 3965 (CR)*	Radula
P. polytrichum C.DC. (2)	Costa Rica	E.J. Tepe	*E.J. Tepe 3470 (CR)*	Radula
P. pseudofuligineum C.DC.	Costa Rica	E.J. Tepe	*E.J. Tepe 3499 (CR)*	Radula
P. pseudogaragaranum Trel.	Panamá	E.J. Tepe	*E.J. Tepe 4041 (PMA)*	Radula
P. sancti-felicis Trel.	Costa Rica	E.J. Tepe	*E.J. Tepe 3415 (CR)*	Radula
P. schuppii A.H. Gentry	Ecuador	E.J. Tepe	*E.J. Tepe 1562 (QCNE)*	Radula
P. silvivagum C.DC.	Costa Rica	E.J. Tepe	*E.J. Tepe 3523 (CR)*	Radula
P. sp	Panamá	E.J. Tepe	*E.J. Tepe 3974 (PMA)*	Radula
P. tectoniifolium Kunth	Brazil	M. Kato	*M. Kato K-1958 (SPF)*	Churumayu
P. tecumense Trel.	Panamá	E.J Tepe	*E.J. Tepe 4008 (PMA)*	Schilleria
P. tuberculatum Jacq.	Panamá	E.J. Tepe	*E.J. Tepe 4039 (PMA)*	Hemipodion
P. umbellatum L.	Panamá	E.J. Tepe	*E.J. Tepe 3967 (PMA)*	Pothomorphe
P. vicosanum Yunck.	Brazil	M. Kato	*M. Kato K-1966 (SPF)*	Isophyllon
P. villalobosense Yunck.	Ecuador	E.J. Tepe	*E.J. Tepe 2952 (QCNE)*	Radula
P. villiramulum C.DC.	Panamá	E.J. Tepe	*E.J. Tepe 4031 (PMA)*	Radula
P. xanthostachyum C.DC.	Costa Rica	E.J. Tepe	*E.J. Tepe 3542 (CR)*	Radula
P. zacatense C.DC.	Costa Rica	E.J. Tepe	*E.J. Tepe 3438 (CR)*	Radula

Table S2. Combined evidence from ^{1}H NMR, GC-MS, LC-MS, LC-UV, and in few cases, 2D-NMR was used to assign metabolite classes to each species. This table includes relevant high-resolution masses, nominal and fragment masses and NMR chemical shifts that were used to classify the compounds present in each individual. In cases where published literature values provided structural confirmation, those reference(s) are listed.

Voucher (herbarium)	Species	Categorical observations	References
E.J. Tepe 3005 (QCNE)	*P. barbatum*	Crude ^{1}H NMR analysis supports the presence of a dihydropyridone ring of piplartine and a minor analog. The major component indicated by ^{1}H NMR is 3,4,5-trimethoxycinnamic acid methyl ester. GC and LC-MS conforms with the ^{1}H NMR analysis, with the major component as the methyl ester along with the carboxylic acid. Additionally, this analysis supports the presence of dihydropiplartine.	[1]
M. Kato K-1958 (SPF)	*P. tectoniifolium*	Crude ^{1}H NMR analysis is consistent with the two *meso*-stereoisomers of grandisin as the major components, with the all syn isomer as the major of the two stereoisomers. Chemical shift analysis is consistent with Kato, et al and other reported values for grandisin. GC-MS analysis confirms the presence of the grandisin stereoisomers.	[2]
E.J. Tepe 3463 (CR)	*P. crassinervium*	Crude ^{1}H NMR analysis is consistent with data reported by Kato et al in previous studies of the natural products chemistry of *P. crassinervium*. The mixture is dominated by the known dihydroquinone, with minor amounts of crassinervic acid, its analogs, and other oxidized prenylated benzoic acid derivatives.	[3-6]
M. Kato K-1954 (SPF)	*P. crassinervium*	The mixture is dominated by crassinervic acid, which was identified by crude ^{1}H NMR analysis and overlapped with the NMR data reported in the literature. Additional analysis by GC-MS confirmed the presence of crassinervic acid as the major component along with other oxidized PBAs that have been previously isolated from *P. crassinervium*. GC-MS analysis confirmed the presence of the flavone sakuranetin, whose fragmentation and molecular ion were consistent with that reported in the NIST database.	[3-6]
E.J. Tepe 4039 (PMA)	*P. tuberculatum*	Characteristic resonances in the crude ^{1}H NMR spectrum indicated the presence of piplartine, which is consistent with literature data for this compound and previous natural product studies of *P. tuberculatum*. LC-MS analysis confirmed the presence of piplartine as well as other minor piper amides (piperolyene and dihydropiperolyene), with other minor long-chain isobutyl amides. GC-MS analysis confirms the presence of piplartine (major), 4,5-Dihydropiperlonguminine, desmethoxypiplartine, and another piperonal derived amide (m/z = 287 with a 135 bp).	[7,8]
E.J. Tepe 3999 (PMA)	*P. cenocladum*	Piplartine, cenocladamide, and sintenpyridone all confirmed by GC-MS and supported by characteristic resonances in the ^{1}H NMR spectrum that overlap with values reported in previous studies of the natural product chemistry of *P. cenocladum*. GC-MS analysis matches that originally reported for this species.	[9]
E.J. Tepe 3956 (PMA)	*P. fimbriulatum*	Crude LC-MS analysis reveals 3 major peaks that are consistent with the bis-furan neolignans diayangambin, sesartemin, and an isomeric analog. ^{1}H-NMR resonances in the crude extract are consistent with the presence of these types of neolignans.	[10-14]

E.J. Tepe 3966 (PMA)	*P. hartwegianum*	^{1}H NMR shows resonances characteristic of piplartine-like amides. LC-MS confirms piplartine as major component of the mixture along with flavonoids/flavones that are supported by ^{1}H NMR analysis. LC-MS analysis also indicates the presence of minor piplartine analogs and long chain piperonal type amides.	
E.J. Tepe 4196 (CR)	*P. holdridgeanum*	Holdrigeanic acid (a prenylated benzoic acid derivative) was found as the major compound that was isolated and characterized using 2D-NMR analysis. LC-MS analysis of the crude extract indicated a variety of other long-chain amides along with the holdrigeanic acid.	Jeffrey and Oliveira, unpubl. data
E.J. Tepe 3034 (QCNE)	*P. maranyonense*	Resonances of 4-nerolidylcatechol type phenol are clearly apparent in the NMR and indicates this as the major component with minor amounts of related compounds. LC-MS analysis supports the presence of a methylated 4-nerolidylcatechol along with other related components.	[15-19]
E.J. Tepe 4069 (PMA)	*P. carrilloanum*	Crude ^{1}H NMR analysis indicates the presence of eupomatenoid-like lignans with evidence of terminal double bonds, many oxygenated, CHOR, Methoxy, methyl doublets. LC-MS is consistent with the presences of an array of these lignans.	[20-26]
E.J. Tepe 4008 (PMA)	*P. tecumense*	Very complex ^{1}H NMR spectrum indicative of a mixture of (neo)lignans, which is supported by a variety of peaks with the molecular ion of m/z = 416 in the LC-MS and the GC-MS fragmentation patterns. The presence of methyl doublets and a highly complex aromatic region is supportive of this conclusion. Sodium adduct is more apparent in the LC-MS, which is a common observation in the ESI-MS analysis of neolignans that are clusin/cubeba like. Masses related to compounds isolated from *P. clusii* and *P. cubeba.*	[27-29]
M. Kato K-1964 (SPF)	*P. goesii*	Crude ^{1}H NMR analysis indicates characteristic lignan resonances with LC-MS and GC-MS indicating (-)-5''-Methoxyhinokinin (MW = 384) isolated from *P. cubeba* and *P. trichostachyon.*	[30]
E.J. Tepe 4044 (PMA)	*P. amphioxys*	Crude ^{1}H NMR analysis indicates characteristic lignan resonances with LC-MS and GC-MS indicating similar patterns to other lignan rich *Piper* species with the observation of peaks with m/z = 296, 372, 326, 356, and 374.	
E.J. Tepe 3961 (PMA)	*P. changuinolanum*	^{1}H NMR analysis suggests the presence of 2-4 sets of tri-substituted aromatic groups with prenyl alkene and methyl resonances suggests a mixture of prenylated benzoic acid derivatives. Additional downfield resonances at 8.5 ppm are characteristic of the oxidized type of PBA similar to resonances found *ortho* to the phenol in methyl tabogonate. LCMS confirms a complex mixture of prenylated phenols along with their oxidized derivatives and GC- and LC-MS indicate the presence of minor flavonoids.	[4, 31-33]
E.J. Tepe 3499 (CR)	*P. pseudofuligineum*	^{1}H NMR indicates flavones, with two trisubstituted aromatics, a cyclized methyl tabagonate, and its carboxylic acid derivative (LC-MS, m/z = 220.22 and 234.25). A minor prenylated flavanone based upon ^{1}H NMR evidence and mass defect in the HRMS is also present.	[4, 31-33]

E.J. Tepe 3963 (PMA)	*P. dryadanum*	^{1}H NMR analysis and GC-MS shows almost exclusively grandisin, with the *anti*-C2 symmetric stereochemistry that is consistent with the NMR resonances reported in the literature.	[2]
E.J. Tepe 1562 (QCNE)	*P. schuppii*	GC-MS suggests sesquiterpenes and a flavanone by comparisons to other GC-MS spectra and the NIST database.	
E.J. Tepe 3534 (CR)	*P. arcteacuminatum*	GC-MS and LC-MS indicate phenethylamine or dihydrochalcone type piper amides (m/z = 288). ^{1}H NMR is consistent with this analysis, clearly showing two sets of triplets of ethylene system and aromatic resonances corresponding to a benzamide or cinnamate type of mono-substitution. These compounds are similar to the altamide/tembamide amides that have been previously isolated from *Piper*.	[34]
E.J. Tepe 3542 (CR)	*P. xanthostachyum*	^{1}H NMR analysis is consistent with the presence of flavonoids, and this is supported by GC-MS and LC-MS analysis, which indicates 4 major flavonoid components. Clear *para*-hydroxybenzyl fragmentation is indicated in the GC-MS of the flavonoids. Some indication of minor amides in the LC-MS.	
E.J. Tepe 3541 (CR)	*P. cyphophyllum*	^{1}H NMR analysis is consistent for the presence of a mixture of lignans with methyl doublets and complexity in the oxygenated and aromatic regions of the crude ^{1}H NMR spectrum. LC-MS is consistent with lignans and/or isoprenylated flavanoids, however NMR is consistent with lignans.	
E.J. Tepe 4041 (PMA)	*P. pseudogaragaranum*	LC-MS and crude ^{1}H NMR is consistent with benzoylated tyramine (phenethylamides) with dihydrochalcones. Dihyrdoxymethoxydihydrochalcones (2) and tyramine with a methoxybenzoyl group.	[34]
E.J. Tepe 2952 (QCNE)	*P. villalobosense*	^{1}H NMR cleanly shows two major compounds with the LC-MS demonstrating two major components [m/z = 311.1785 (M+H) and 297.1626 (M+H)]. ^{1}H-NMR MS are consistent for two related bis amides *N,N'*-dibenzoylputricene and *N,N'*-dibenzoylcadavine, which have been isolated previously from *Haplophyllum*.	[35-38]
E.J. Tepe 3433 (CR)	*P. peracuminatum*	^{1}H NMR clearly shows a prenylated and diprenylated dihydrochalcone that were confirmed by isolation and full 2D spectroscopic characterization. LC-MS analysis confirms the presence of these dihydrochalcone derivatives.	Jeffrey and Oliveira, unpubl. data
E.J. Tepe 3502 (CR)	*P. colonense*	Two chalcones (one major: one minor) are clearly indicated in the ^{1}H NMR spectrum the LC- and GC-MS with their corresponding flavanones. Sakuranetin is present in the GC- and LC-MS analysis and confirmed by the NIST database. ^{1}H NMR analysis also indicates one prenyl group that is consistent with the presence of methyl 3-prenyl-4-hydroxybenzoate in the LC/GC-MS. Clear diastereotopic ABX pattern indicative of a dihydroflavone as one of the major components.	

E.J. Tepe 4062 (PMA)	*P. peracuminatum*	^{1}H NMR analysis clearly shows resonances that are characteristic of *trans*-double bonds (J ~ 15 Hz). Additional complexity across the downfield and upfield regions of the spectrum with a clear CH_2CH_2 phenethyl structure indicated by two triplets. Cinnamoyl fragmentation in the GC- and LC-MS suggests a mixture of cinnamylphenethylamides related to those that have been isolated from other species of *Piper*.	
E.J. Tepe 3413 (CR)	*P. culebranum*	LC- and GC-MS provide evidence of two chalcones that are identical to those found in *P. colonense* (m/z = 314 and 270) along with the corresponding flavanones, and sakuranetin. Neutral loss of water from m/z = 284 indicating hydroxyflavone of m/z = 256.	
E.J. Tepe 4032 (PMA)	*P. colonense*	2 chalcones. Clear diastereotopic ABX pattern indicative of a dihydroflavone as one of the major components. Prenyl alkene resonance is consistent with the presence of methyl 3-prenyl-4-hydroxybenzoate. 2 chalcones (one major: one minor) with their corresponding flavanones, including sakuranetin.	
E.J. Tepe 3527 (CR)	*P. culebranum*	Methyl 3-prenyl-4-hydroxybenzoate is clearly present in the LC-MS and is supported in the ^{1}H NMR with one prenylated alkene resonance. Other peaks correspond to the presence of two chalcones. Clear diastereotopic ABX pattern indicative of a dihydroflavone as one of the major components. Prenyl checks out with methyl 3-prenyl-4-hydroxybenzoate. 2 chalcones (one major: one minor) with their corresponding flavanones. Sakuranetin.	
E.J. Tepe 4005 (PMA)	*P. culebranum*	^{1}H NMR analysis supports the presence of methyl tabagonate and its methyl ether, which is further supported in the LC/GC-MS with m/z = 314, 270, 284. Additional chalcones and flavanones present.	[4, 31-33]
E.J. Tepe 131 (CR)	*P. friedrichsthalii*	Spectroscopic analysis consistent with a cyclized (chromanone) of methyl tabagonate and an oxidized tabagonate with a third related compound.	[4, 31-33]
E.J. Tepe 3036 (QCNE)	*P. longicaudatum*	LC-MS yielded a peak that corresponded to the mass of anofinic acid methyl ester ($[M+H]^+$ = 235.0997) that could not be confirmed by NMR due to low signal. Anofinic acid occurs naturally in mushrooms and is similar in structure to gaudichaudianic acid Stigmasterol. Sitosterol are all apparent in the GC-MS with 2-aminosteroids/triterpenes (C30+NH2). The aminosteroid mass was confirmed by LC-MS: $[M+H]^+$ = 420.3503.	[39]
M. Kato K-1960 (SPF)	*P. chimonanthifolium*	LC-MS is indicative of the presence of glycosylated. There are some interesting protons in the 6.2-6.9 range that could suggest chromenes or a styrenyl alkene. GC-MS indicates m/z = 284, 256, which are supportive of the presence of 5,7-dihydroxyflavanone and wogonin.	
M. Kato K-1942 (SPF)	*P. mollicomum*	NMR is low signal, however LC-MS shows the acid aduncum chromene, dihydroxyp-methoxy dihydrochalcone, piperaduncin A, and piperaduncin B.	[40]

M. Kato K-1978 (SPF)	*P. aduncum* var. *cordulatum*	LC-MS flavanoid glycosides, which is consistent with the ^{1}H NMR data. Dihydrochalcones similar to what has been previously isolated from *Piper aduncum* are indicated. Additional, chromene type structures that are related to aduncum chromenes and their oxidized derivatives.	[41-44]
M. Kato K-1983 (SPF)	*P. gaudichaudianum*	Spectroscopic analysis consistent with compounds previously reported from *Piper gaudichaudianum.*	[4, 31, 45]
E.J. Tepe 4394 (USM)	*P. armatum*	The NMR and GC-MS suggests a benzoylamide similar to the phenylalanine peptides that have been isolated previously from *Piper aurantiamide* A and B. Significant ^{1}H NMR resonance overlap with these reports. LC-MS indicates a m/z = 253, sometimes 254 could be a fragment.	[46]
E.J. Tepe 4393 (USM)	*P. chanchamayanum*	The phytochemical profile was surprisingly similar to that of *P. disparipes*, having all three of phenolic glycosides found there.	
E.J. Tepe 3426 (CR)<br>*E.J. Tepe 3486 (CR)*<br>*E.J. Tepe 3543 (CR)*	*P. disparipes*	An early eluting peak (0.6 min) with a mass defect suggesting a phenolic glycoside ($[M+H]^+$ = 543.1347), a peak at 0.75 min that had a mass suggesting a trihydroxy flavanone glycoside (METLIN match, $[M+H]^+$ = 565.1627), and a peak at 0.9 min that had a mass suggesting a tetrahydroxy flavanone (such as kaempferol) glycoside (METLIN match, $[M+H]^+$ = 579.1777). These predominant flavonoid glycosides were found in all three *P. disparipes* individuals, which all had very similar chemistry. These proposed classes could not be confirmed by ^{1}H NMR.	
E.J. Tepe 3430 (CR)	*P. hispidum*	7+ flavonoids are clearly indicated in the GC-MS and is additionally supported by the ^{1}H NMR spectrum.	
E.J. Tepe 3523 (CR)	*P. silvivagum*	We found a peak that was attributed to anofinic acid methyl ester in *P. longicaudatum.* Both the retention time (5.8 min) and *m/z* matched. We also found a mass that corresponded to trihydroxy biphenyl structure (METLIN match; $[M+H]^+$ = 203.0729), which was categorized as a phenyl propanoid, and two aliphatic amides (METLIN match; $[M+H]^+$ = 404.3599, 446.3683).	
A.E. Glassmire YY1 (CINC)	*P. baezense*	GC-MS clearly indicates a series of 4-alkenyl phenols: 2 dienyl-C12; 1-C12 monoalkenyl, and 1-C14 alkenyl along with polyunsaturated fatty acids. ^{1}H NMR is characteristic for the presence of alkenyl phenols.	[47]
E.J. Tepe 3476 (CR)	*P. hispidum*	Two major flavonoids indicated by GC-MS, and this is clearly supported in the crude ^{1}H NMR spectrum.	
M. Kato 1948 (SPF)	*P. mosenii*	^{1}H NMR and MS were inconclusive, although tabogenates have been found in the literature, they were not found in our analysis.	[33]
M. Kato K-1945 (SPF)	*P. malacophyllum*	^{1}H NMR and MS are consistent with *p*-alkenyl phenols that have been previously isolated from this species. LC-MS and NMR confirm the presence of gibbilimbol.	[48]

M. Kato K-1944 (SPF)	*P. lagoense*	^{1}H NMR analysis indicates chromene type peaks similar to those isolated from *Piper kelleyii.* LC-MS indicates isobaric compounds to the *kelleyii* chromene/guachaudianic acid, oxidation products. A farnesylated hydroxy benzoic acid derivative is also indicated. Dimeric compounds and oxidized dimeric compounds are indicated in the LC-MS.	
M. Kato K-1949 (SPF)	*P. gaudichaudianum*	Gaudichaudianic acid and related prenylated compounds are clearly indicated by LC-MS and ^{1}H NMR analysis.	[4]
E.J. Tepe 3993 (PMA)	*P. lucigaudens*	Low signal in the NMR, however, there are peaks that align with pyrone kava lactones of 5,6-dehydrokavain or desmethoxyyangonin and its dimer (perhaps [2+2]). Additional flavonoids are indicated in the GC/LC-MS.	[49]
E.J. Tepe 4031 (PMA)	*P. villiramulum*	^{1}H NMR and GC-MS analysis for the presence of *p*-alkenylphenols that have been previously isolated from this species.	[50]
E.J. Tepe 3965 (CR)	*P. polytrichum*	^{1}H NMR and GC-MS indicates the presence of three major alkenyl phenols, m/z = 344, 314 (diene) along with fatty acids related to those previously isolated from *Piper* species.	[48]
E.J. Tepe 3482 (CR)	*P. chrysostachyum*	^{1}H NMR and GC-MS indicates the presence of a series of alkenyl phenols.	[47]
E.J. Tepe 3415 (CR)	*P. sancti-felicis*	GC-MS and LC-MS are both consistent with a mixture chalcones, dihydrochalcones, and flavonoids, with some mix of methylation. ^{1}H NMR is consistent with this analysis with distinct peaks for the chalcone alkene and a mono-substituted aromatic.	
E.J. Tepe 3496 (CR)	*P. hispidum*	GC-MS confirms the presence of *p*-alkenylphenols with additional unsaturated fatty acids.	
E.J. Tepe 4028 (PMA)	*P. lucigaudens*	LC-MS analysis indicates chromenes and related dimers that are isobaric to the *P. kelleyi* system. ^{1}H NMR is consistent with the presence of these type of compounds.	[49]
E.J. Tepe 3537 (CR)	*P. hispidum*	Major compound (m/z =275, M+H) and ^{1}H NMR are nearly identical to 3509 and 3974. Resonances match for a butenolide or kava-type lactone that has been previously isolated from *Piper sanctum.*	[51]
E.J. Tepe 3509 (CR)	*P. hispidum*	Nearly identical to *P. hispidum E.J. Tepe 3537 (CR).*	[51]
E.J. Tepe 3965 (CR)	*P. polytrichum*	^{1}H NMR and GC-MS analysis clearly indicate the presence of a series of *p*-alkenylphenols that are related to those previously isolated from *Piper* species.	[47]
E.J. Tepe 3974 (PMA)	*P.* sp.	Spectra are nearly identical to those of *P. hispidum E.J. Tepe 3537 (CR)* and *P. hispidum E.J. Tepe 3509 (CR).*	[51]

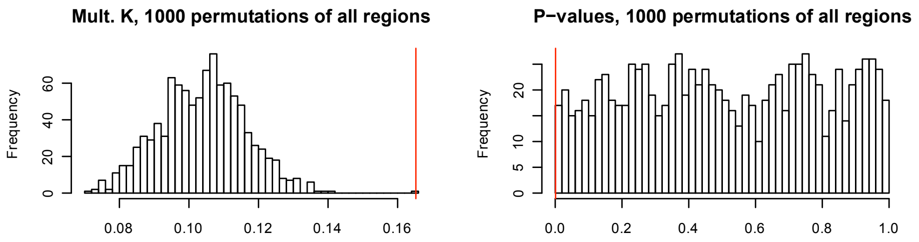

Figure S1: Results of the multivariate K test on 1000 permutations of all chemical regions indicate that our observed, significant phylogenetic signal is not an artifact of zero inflation exhibited by the ^{1}H NMR data. Vertical red lines represent our observed values for the multivariate K statistic and its associate *P*-value.

Supplementary References

1. Duh, C. Y., Wu, Y. C. & Wang, S. K. Cytotoxic pyridine alkaloids from *Piper aborescens*. *Phytochemistry* **29**, 2689-2691 (1990).
2. Ramos, C. S. *et al.* Configuration and stability of naturally occurring all-*cis*-tetrahydrofuran lignans from *Piper solmsianum*. *RSC Adv.* **7**, 46932-46937 (2017).
3. Danelutte, A. P., Lago, J. H. G., Young, M. C. M., & Kato, M. J. Antifungal flavanones and prenylated hydroquinones from *Piper crassinervium* Kunth. *Phytochemistry* **64**, 555-559 (2003).
4. Lago, J. H. G. *et al.* Benzoic acid derivatives from *Piper* species and their fungitoxic activity against *Cladosporium cladosporioides* and *C. sphaerospermum*. *J. Nat. Prod.* **67**, 1783-1788 (2004).
5. Lopes, A. A. *et al. In vitro* activity of compounds isolated from *Piper crassinervium* against *Trypanosoma cruzi*. *Nat. Prod. Res.* **22**, 1040-1046 (2008).
6. Yamaguchi, L. F., Lago, J. H. G., Tanizaki, T. M., Di Mascio, P., & Kato, M. J. Antioxidant activity of prenylated hydroquinone and benzoic acid derivatives from *Piper crassinervium* Kunth. *Phytochemistry* **67**, 1838-1843 (2006).
7. Chaves, M. C. O., Júnior, A. G. F., & Santos, B. V. O. Amides from *Piper tuberculatum* fruits. *Fitoterapia* **74**, 181-183 (2003).
8. da Silva, R. V. *et al.* Antifungal amides from *Piper arboretum* and *Piper tuberculatum*. *Phytochemistry* **59**, 521-527 (2002).
9. Dodson, C. D., Dyer, L. A., Searcy, J., Wright, Z., & Letourneau, D. K. Cenocladamide, a dihydropyridone alkaloid from *Piper cenocladamide*. *Phytochemistry* **53**, 51-54 (2000).
10. Ahmed, A. A. *et al.* Two highly oxygenated eudesmanes and 10 lignans from *Achillea holosericea*. *Phytochemistry* **59**, 851-856 (2002).
11. Mota, J. d. S. *et al. In vitro* trypanocidal activity of phenolic derivatives from *Peperomia obtusifolia*. *Planta Med.* **75**, 620-623 (2009).
12. Rios, M. Y., Ocampo-Acuña, Y. D., Ramírez-Cisneros, M. A., & Salazar-Rios, M. E. Furofuranone lignans from *Leucophyllum ambiguum*. *J. Nat. Prod.* **83**, 1424-1431 (2020).
13. Shao, S.-Y., Yang, Y.-N, Feng, Z.-M., Jiang, J.-S., & Zhang, P.-C. An efficient method for determining the relative configuration of furofuran lignans by ^{1}H NMR spectroscopy. *J. Nat. Prod.* **81**, 1023-1028 (2018).
14. Solís, P. N. *et al.* A new larvicidal lignan from *Piper fimbriulatum*. *Pharm. Biol.* **43**, 378-381 (2005).
15. Bagatela, B. S. *et al.* Evalution of antimicrobial and antimalarial activities of crude extract, fractions and 4-nerolidylcathecol from the aerial parts of *Piper umbellata* L. (Piperaceae). *Nat. Prod. Res.* **27**, 2202-2209 (2013).
16. Baldoqui, D. C., Bolzani, V. d. S., Furlan, M., Kato, M. J., & Marques, M. O. Flavones, lignans, and terpene from *Piper umbellata* (Piperaceae). *Quím. Nova* **32**, 1107-1109 (2009).
17. Kijjoa, A., Giesbrecht, A. M., Akisue, M. K., Gottlieb, O. R., & Gottlieb, H. E. 4-nerolidylcatechol from *Potomorphe umbellata*. *Planta Med.* **39**, 85-87 (1980).
18. da Silva Pinto, A. C. *et al. Piper peltatum*: biomass and 4-nerolidylcatechol production. *Planta Med.* **76**, 1473-1476 (2010).
19. Tabopda, T. K. *et al.* Bioactive aristolactams from *Piper umbellatum*. *Phytochemistry*, **69**, 1726-1731 (2008).

20. Carinin, M., Aldini, G., Orioli, M., & Facino, R. M. Antioxidant and photoprotective activity of a lipophilic extract containing neolignans from *Krameria triandra* roots. *Planta Med.* **68**, 193-197 (2002).
21. Freixa, B., Vila, R., Ferro, E. A., Adzet, T., & Cañigueral, S. Antifungal principles from *Piper fulvescens*. *Planta Med.* **67**, 873-875 (2001).
22. Johann, S. *et al.* Antifungal activities of compounds isolated from *Piper abutiloides* Kunth. *Mycoses* **52**, 499-506 (2009).
23. Macedo, A. L. *et al.* Isolation of a larvicidal compound from *Piper solmsianum* C.DC. (Piperaceae). *Nat. Prod. Res.* **32**, 2701-2704 (2018).
24. Marçal, F. J. B., Cortez, D. A. G., Ueda-Nakamura, T., Nakamura, C. V., & Dias Filho, B. P. Activity of the extracts and neolignans from *Piper regnellii* against methicillin-resistant *Staphylococcus aureus* (MRSA). *Molecules* **15**, 2060-2069 (2010).
25. Pessini, G. L., Dias Filho, B. P., Nakamura, C. V., Ferreira, A. G., & Cortez, D. A. G. Neolignans and the analysis of the essential oil of *Piper regnellii* (Miq.) C. DC. var. *pallescens* (C. DC.) Yunck leaves. *Rev. Bras. Farmacogn.* **15**, 199-204 (2005).
26. Scodro, R. B. L. *et al.* Anti-tuberculosis neolignans from *Piper regnellii*. *Phytomedicine*, **20**, 600-604 (2013).
27. Badheka, L. P., Prabhu, B. R., & Mulchandani, N. B. Dibenzylbutyrolactone lignans from *Piper cubeba*. *Phytochemistry*, **25**, 487-489 (1986).
28. Badheka, L. P., Prabhu, B. R., & Mulchandani, N. B. Lignans of *Piper cubeba*. *Phytochemistry* **26**, 2033-2036 (1987).
29. Prabhu, B. R., & Mulchandani, N. B. Lignans from *Piper cubeba*. *Phytochemistry* **24**, 329-331 (1985).
30. Koul, S. K., Taneja, S. C., Pushpangadan, P., & Dhar, K. L. Lignans of *Piper trichostachyon*. *Phytochemistry* **27**, 1479-1482 (1988).
31. Gaia, A. M., Yamaguchi, L. F., Jeffrey, C. S., & Kato, M. J. Age-dependent changes from allylphenol to prenylated benzoic acid production in *Piper gaudichaudianum* Kunth. *Phytochemistry* **106**, 86-93 (2014).
32. Terreaux, C., Gupta, M. P., & Hostettmann, K. Antifungal benzoic acid derivatives from *Piper dilatatum*. *Phytochemistry* **49**, 461-464 (1998).
33. Zermiani, T. *et al.* Morphological and phytochemical characterization of *Piper mosenii. Nat. Prod. Commun.* **14**, 1934578X1901400118 (2019).
34. Maxwell, A., & Rampersad, D. β-phenylethylamine-derived amides from *Piper guayranum. J. Nat. Prod.* **52**, 411-414 (1989).
35. Nesmelova, E. F., Bessonova, I. A., & Yunusov, S. Y. Haplamidine – a new alkaloid from *Haplophyllum latifolium*. *Khim. Prir. Soedin.* **3**, 427 (1977).
36. Nesmelova, E. F., Bessonova, I. A., & Yunusov, S. Y. The structure of haplamide and the synthesis of haplobucharine. *Khim. Prir. Soedin.* **2**, 289 (1977).
37. Nesmelova, E. F., Bessonova, I. A., & Yunusov, S. Y. Alkaloids of *Haplophyllum latifolium*. Structure of haplatine. *Khim. Prir. Soedin.* **6**, 758-764 (1978).
38. Nesmelova, E. F., Bessonova, I. A., & Yunusov, S. Y. Alkaloids of *Haplophyllum latifolium*. Structure and synthesis of haplamide and haplamidine. *Khim. Prir. Soedin.* **6**, 749-752 (1978).
39. Tan, R. X., Wolfender, J.-L., Ma, W. G., Zhang, L. X., & Hostettmann, K. Secoiridoids and antifungal aromatic acids from *Gentiana algida*. *Phytochemistry* **41**, 111-116 (1996).

40. Orjala, J., Wright, A. D., Behrends, H., Folkers, G., & Sticher, O. Cytotoxic and antibacterial dihydrochalcones from *Piper aduncum*. *J. Nat. Prod.* **57**, 18-26 (1994).
41. Baldoqui, D. C. *et al.* A chromene and prenylated benzoic acid from *Piper aduncum*. *Phytochemistry* **51**, 899-902 (1999).
42. Lago, J. H. G. *et al.* Prenylated benzoic acid derivatives from *Piper aduncum* L. and *P. hostmannianum* C. DC. (Piperaceae). *Phytochem. Lett.* **2**, 96-98 (2009).
43. Moreira, D. d. L., Guimarães, E. F., & Kaplan, M. A. C. A chromene from *Piper aduncum*. *Phytochemistry* **48**, 1075-1077 (1998).
44. Orjala, J., Erdelmeier, C. A. J., Wright, A. D., Rali, T., & Sticher, O. Two chromenes and a prenylated benzoic acid derivative from *Piper aduncum*. *Phytochemistry*, **34**, 813-818 (1993).
45. Batista, J. M. *et al.* Absolute configuration and selective trypanocidal activity of gaudichaudianic acid enantiomers. *J. Nat. Prod.* **74**, 1154-1160 (2011).
46. Banerji A., & Ray, R. Aurantiamides: a new class of modified dipeptides from *Piper aurantiacum. Phytochemistry* **20**, 2217-2220 (1981).
47. Maynard, L. D. *et al.* Secondary metabolites in a neotropical shrub: spatiotemporal allocation and role in fruit defense and dispersal. *Ecology* **101**, e03192 (2020).
48. Yoshida, N. C., *et al.* Alkenylphenols from *Piper dilatatum* and *P. diospyrifolium. Phytochem. Lett.* **25**, 136-140 (2018).
49. Jeffrey, C. S. *et al.* Antiherbivore prenylated benzoic acid derivatives from *Piper kelleyi*. *J. Nat. Prod.* **77**, 148-153 (2014).
50. Galinis, D. L., & Wiemer, D. F. Villiramulins A and B: new phenol derivatives from *Piper villiramulum. J. Org. Chem.* **58**, 7804-7807 (1993).
51. Pelter, A., & Hansel, R. Epoxipiperolid from *Piper sanctum*. *Z. Naturforsch B* **27**, 1186-1190 (1972).
